# Association between recently raised anticholinergic burden and risk of acute cardiovascular events: nationwide case-case-time-control study

**DOI:** 10.1136/bmj-2023-076045

**Published:** 2023-09-27

**Authors:** Wei-Ching Huang, Avery Shuei-He Yang, Daniel Hsiang-Te Tsai, Shih-Chieh Shao, Swu-Jane Lin, Edward Chia-Cheng Lai

**Affiliations:** 1 School of Pharmacy, Institute of Clinical Pharmacy and Pharmaceutical Sciences, College of Medicine, National Cheng Kung University, Tainan, Taiwan; 2 Department of Pharmacy, Keelung Chang Gung Memorial Hospital, Keelung, Taiwan; 3 Department of Pharmacy Systems, Outcomes & Policy, College of Pharmacy, University of Illinois at Chicago, Chicago, IL, USA

## Abstract

**Objective:**

To evaluate the association between recently raised anticholinergic burden and risk of acute cardiovascular events in older adults.

**Design:**

Case-case-time-control study (ie, incorporating a case crossover design and a control crossover design consisting of future cases).

**Setting:**

Taiwan’s National Health Insurance Research Database.

**Participants:**

317 446 adults aged ≥65 who were admitted to hospital because of an incident acute cardiovascular event between 2011 and 2018. Acute cardiovascular events included myocardial infarction, strokes, arrhythmias, conduction disorders, and cardiovascular death.

**Main outcome measures:**

The anticholinergic burden was measured for each participant by adding up the anticholinergic scores for individual drugs using the Anticholinergic Cognitive Burden Scale. Scores were classified into three levels (0 points, 1-2 points, and ≥3 points). For each participant, anticholinergic burden levels during hazard periods (day −1 to −30 before the cardiovascular event) were compared with randomly selected 30 day reference periods (ie, periods between days −61 and −180). Conditional logistic regression determined odds ratios with 95% confidence intervals to evaluate the association between acute cardiovascular events and recently raised anticholinergic burden.

**Results:**

The crossover analyses included 248 579 current cases. Participants’ average age on the index date was 78.4 years (standard deviation 0.01), and 53.4% were men. The most frequently prescribed drugs with anticholinergic activity were antihistamines (68.9%), gastrointestinal antispasmodics (40.9%), and diuretics (33.8%). Among patients with varying levels of anticholinergic burden in different periods, more patients carried higher levels of anticholinergic burden during hazard periods than during reference periods. For example, 17 603 current cases had 1-2 points of anticholinergic burden in the hazard period with 0 points in the reference period, while 8507 current cases had 0 points in the hazard period and 1-2 points in the reference period. In the comparison of 1-2 points versus 0 points of anticholinergic burden, the odds ratio was 1.86 (95% confidence interval 1.83 to 1.90) in the case crossover analysis and 1.35 (1.33 to 1.38) in the control crossover analysis, which yielded a case-case-time-control odds ratio of 1.38 (1.34 to 1.42). Similar results were found in the comparison of ≥3 versus 0 points (2.03, 1.98 to 2.09) and ≥3 versus 1-2 points (1.48, 1.44 to 1.52). The findings remained consistent throughout a series of sensitivity analyses (eg, cut-off points for anticholinergic burden categories were redefined and different scales were used to measure anticholinergic burden).

**Conclusions:**

An association was found between recently raised anticholinergic burden and increased risk of acute cardiovascular events. Furthermore, a greater increase in anticholinergic burden was associated with a higher risk of acute cardiovascular events.

## Introduction

Ageing populations present various challenges for healthcare worldwide, including increases in multimorbidity rates and subsequent polypharmacy issues. Polypharmacy has been associated with various unintended clinical consequences, with a prevalence reported to be as high as 90% in older adults.^1^ ^2^ ^3^ Drugs with anticholinergic activity are among the most commonly prescribed drugs in older adults with polypharmacy.^4^ ^5^ ^6^ However, for most of these drugs, anticholinergic activity is not the main intended effect and is often considered a side effect. As a result, clinicians might prescribe these drugs without taking into account their anticholinergic activity.^7^ ^8^ Anticholinergic burden refers to the cumulative adverse effect of several drugs with anticholinergic activity.^9^ Some studies have reported an association between anticholinergic burden and several long term adverse outcomes, including acute cardiovascular events such as myocardial infarction, strokes, and cardiovascular death.^10^ ^11^ ^12^ ^13^ ^14^ This research was based on biological plausibility from existing evidence that drugs with anticholinergic activity have pro-arrhythmic and pro-ischaemic effects that can lead to tachyarrhythmias and increased oxygen requirements.^15^

The onset of anticholinergic effects can be rapid after taking a drug, which increases the likelihood of acute cardiovascular events. Research is needed examining the risk of acute cardiovascular events in the light of recently increased anticholinergic burden. Therefore, this study aimed to evaluate the association between recently raised anticholinergic burden and the risk of acute cardiovascular events. Additionally, patients receiving anticholinergic drugs might have several morbidities, leading to possible confounding by indications compared with those not receiving anticholinergic drugs. A clinician might prescribe drugs (such as antivertigo drugs for dizziness) for the symptoms of cardiovascular events before a confirmed diagnosis, making it challenging to infer a causal relation or even leading to reverse causality between drug use and outcomes (that is, protopathic bias).^16^ We performed a case-case-time-control study incorporating two self-controlled analyses—a case crossover analysis and a control crossover analysis consisting of future cases to address confounding by indication and potential protopathic bias, respectively.

## Methods

### Data source

The data were retrieved between 2006 and 2018 from Taiwan’s National Health Insurance Research Database, an administrative claims database associated with Taiwan's National Health Insurance programme, which covers 99.9% of Taiwan’s population (approximately 23 million people). Details of the database have been described elsewhere.^17^ Briefly, the database contains comprehensive records of diagnoses, drug prescriptions and dispensations, procedures, and healthcare use in different healthcare settings such as hospitals, clinics, and contracted community pharmacies. Major disease diagnoses have been validated by previous studies, including acute myocardial infarction,^18^ ischaemic stroke,^19^ haemorrhagic stroke,^20^ hypertension,^21^ heart failure,^22^ and other comorbidities.^17^ ^23^ We linked the database to the National Cause of Death registry to obtain information about the dates and causes of death. The data linkage between the National Health Insurance Research Database and the National Cause of Death Registry was at a high level of completeness.^24^

### Study population

We selected older adults aged ≥65 years with incident acute cardiovascular events that required hospital admission between 1 January 2011 and 31 December 2018. Acute cardiovascular events were defined as myocardial infarction, strokes, dysrhythmias, conduction disorders, syncope, and cardiovascular death, all of which could have been related to the anticholinergic effect of drugs on the cardiovascular system.^15^ ^25^ The events were captured based on admission diagnoses using the international classification of diseases, ninth and tenth revisions, clinical modification (ICD-9-CM and ICD-10-CM), as listed in supplementary table 1. The diagnostic codes recorded during the admissions claims identified the events, comorbidities, and drugs used. We excluded patients with a history of acute cardiovascular events in the five years preceding the event dates.

### Study design

**Fig 1:**
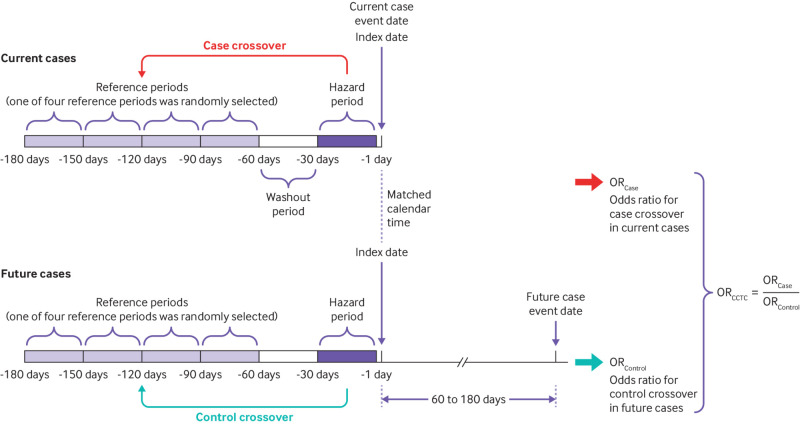
Case-case-time-control (CCTC) design and details of time windows. The case-case-time-control analysis incorporated two self-controlled analyses—a case crossover analysis and a control crossover analysis consisting of future cases to address confounding by indication and potential protopathic bias, respectively

We used a case-case-time-control design in this study, which included two self-controlled analyses—a case crossover analysis and a control crossover analysis consisting of future cases.^16^ ^26^ The case crossover design eliminated time invariant confounders through within-individual comparisons.^27^ ^28^ The control crossover analysis was performed to adjust for time trends in drug use and to address the likely protopathic bias by including future cases.^16^ ^29^ We matched current cases to future cases one-to-one by age and sex. Specifically, for the future cases, we selected patients whose event dates were within 60-180 days after the matched event dates of the current cases. We defined the index dates as the event dates of the current cases and assigned the same index dates for the future cases. We divided the 180 days preceding the index dates into several 30 day intervals, including a hazard period (days −1 to −30), a washout period (days −31 to −60), and a reference period randomly selected from four different 30 day reference periods (between days −61 and −180). Figure 1 presents the case-case-time-control design and details of the time windows.

### Anticholinergic burden measurement

We used the Anticholinergic Cognitive Burden Scale, a well established and widely used tool to measure anticholinergic burden.^30^ Supplementary table 2 lists details of drugs and their scores. Briefly, the scale categorises drugs according to their anticholinergic potency, ranging from 0 points (no anticholinergic potency), to 1 point (mild potency), to 2 and 3 points (high potency). The total anticholinergic burden during each period was calculated as the sum of drugs multiplied by their corresponding points. Preparations used as topical agents were not included in the calculation; however, inhaled drugs were included because previous studies have suggested they might be associated with the risk of cardiovascular events owing to their potential for systemic absorption.^15^ ^31^ We classified scores into three levels (0 points, 1-2 points, and ≥3 points) in line with previous studies.^12^ ^13^ ^14^ Supplementary table 3 presents details of the anticholinergic burden categories.

### Statistical analysis and covariates

We defined the baseline period as one year before the event date during which data on comorbidities and drug use were collected (supplementary tables 4 and 5). To evaluate the risk of acute cardiovascular events associated with anticholinergic burden, we used conditional logistic regression to estimate the odds ratios with 95% confidence intervals for levels of anticholinergic burden, comparing the hazard period with the reference period. We calculated case-case-time-control odds ratios as the odds ratios from the case crossover analysis divided by the odds ratios from the control crossover analysis. Supplementary information 1 gives details about the 95% confidence interval calculation. We considered the composite outcome of acute cardiovascular events, including myocardial infarction, strokes, dysrhythmias, conduction disorders, and cardiovascular death. We also evaluated the risk of each cardiovascular event separately in a subgroup analysis by one-to-one matching of current cases to future cases by diagnoses of acute cardiovascular events in addition to age and sex.

### Sensitivity analysis

We conducted a series of sensitivity analyses to examine the robustness of our results. Firstly, we redefined the cut-off points for anticholinergic burden categories from ≥3 points to ≥5 points and ≥10 points; we redefined the interval of the hazard and reference periods from 30 days to 14 days; and we redefined the time interval of the washout period from 30 days to 60 days and 120 days. We also redefined future cases by selecting patients whose events occurred between 120 and 240 days after the event dates of the current cases. Given that there is no standard definition of at risk duration that could be applied to this study design, our sensitivity analyses aimed to test various assumptions. Secondly, we recalculated the anticholinergic burden by considering the drug dosage using the World Health Organization’s defined daily dose. The dose adjusted anticholinergic burden for each drug was calculated by multiplying the original score by the daily drug dosage.^32^ This method took into account the duration of treatment when calculating the daily drug dosage.

Thirdly, we excluded drugs prescribed for less than three days and less than seven days to ensure sufficient duration of drug use. Fourthly, we tested biases from time varying confounders by following the literature. We considered acute conditions as time varying variables in the regression models, including upper respiratory tract infection, pneumonia, influenza, urinary tract infection, bloodstream infection, endocarditis, myocarditis, and acute kidney injury^33^ ^34^ ^35^ ^36^ ^37^ ^38^ ^39^ (supplementary table 6). Finally, we repeated all analyses using four additional scales measuring the anticholinergic burden: the Anticholinergic Drug Scale,^40^ ^41^ the German Anticholinergic Burden Scale,^42^ the Modified Anticholinergic Cognitive Burden Scale,^43^ and the Korean Anticholinergic Activity Scale.^44^ We selected these scales because they are well established and some of them have been developed in recent years. Additionally, the Modified Anticholinergic Cognitive Burden Scale and the Korean Anticholinergic Activity Scale cover drugs frequently used in Asian countries. Supplementary table 7 presents details of these scales.

**Fig 2:**
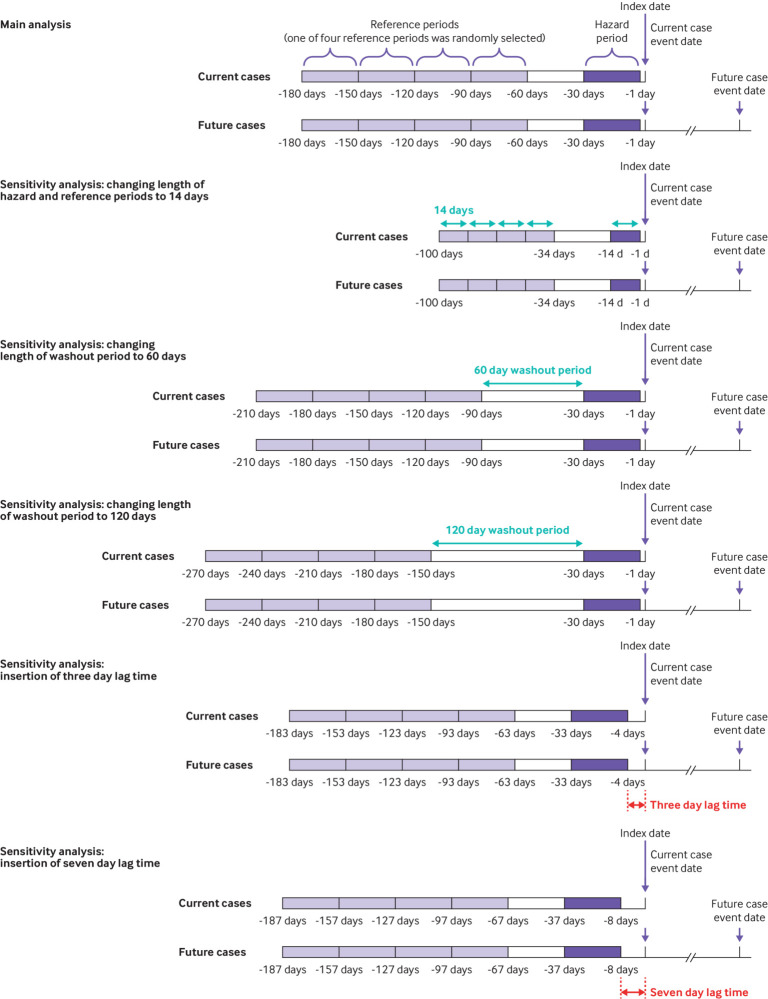
Main analysis and sensitivity analyses based on various time periods

To further address the issue of protopathic bias, we adopted a three day and a seven day lag time between the index date and the hazard period in the analysis to minimise any potential impact. Figure 2 shows the sensitivity analyses based on various time periods. Furthermore, we excluded patients receiving cardiovascular drugs in the year before the index dates to reduce protopathic bias and to address potential underdiagnosis in the database. Supplementary table 2 defines the cardiovascular drugs. Because of the potential protopathic bias resulting from patients receiving several drugs with anticholinergic activity during hospital admission before their death, we conducted sensitivity analyses by excluding patients who had died, and also by excluding those with a record of hospital admission during the baseline period. Furthermore, we performed sensitivity analyses by excluding patients who had myocarditis, endocarditis, or acute infection to address any possible association between these diseases and acute cardiovascular events.^33^ ^35^ ^36^ ^37^ ^38^ ^39^

In addition to the future case control analysis, we selected a group of control patients without acute cardiovascular events between 2011 and 2018 for a non-case control analysis 26 (supplementary figs 1 and 2). We randomly matched non-case control patients to case patients one-to-one based on the same index date and disease risk score, which was the probability of event occurrence in the absence of drug use.^45^ We repeated the analysis using the non-case group for the case-time-control analysis (supplementary information 2). We calculated the disease risk score using measured covariates collected during a one year baseline period. We conducted a sensitivity analysis by measuring the baseline covariates one year before the reference period (from −180 days to −540 days) to avoid adjusting for factors occurring after drug use. Supplementary figure 3 shows a flowchart for the selection of the external control group. Supplementary table 4 liststhe covariates for a disease risk score to estimate the probability of developing the cardiovascular events.

Finally, to minimise potential bias because the database does not include over-the-counter drug data, we performed a more restrictive analysis by selecting people with chronic conditions, including hypertension, diabetes mellitus, or hyperlipidaemia. These patients were more likely to obtain all their drugs from clinicians than from over-the-counter sources.

### Patient and public involvement

No patients or members of the public were directly involved in this study owing to restrictions due to the covid-19 pandemic in Taiwan.

## Results

### Baseline characteristics of study population

**Fig 3:**
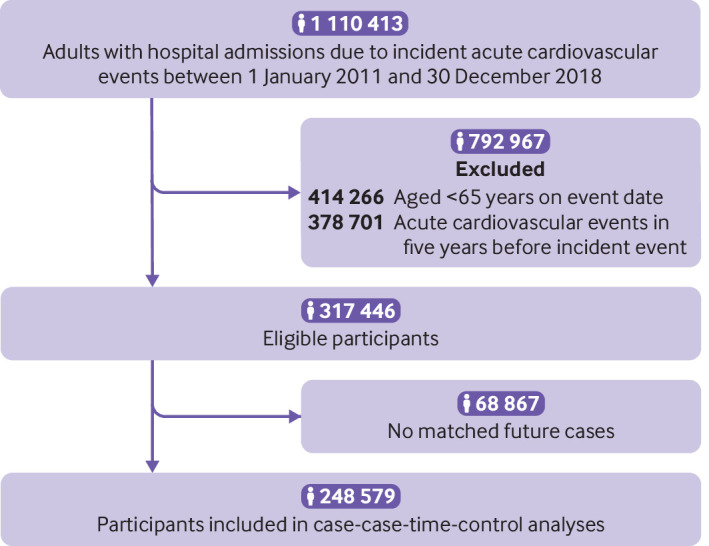
Flowchart of study population and selection of eligible patients

Of 317 446 study participants identified, we included 248 579 current cases in the crossover analyses after one-to-one matching to future cases (fig 3). Table 1 describes the baseline characteristics of the study population and the matched current and future cases. The average age of the study population on the index date was 78.4 years (standard deviation 0.01); 53.4% were men. The most common comorbidities were hypertension (62.2%), followed by diabetes mellitus (32.3%) and dyslipidaemia (24.0%). The most frequently prescribed drugs with anticholinergic activity were antihistamines (68.9%), gastrointestinal antispasmodics (40.9%), and diuretics (33.8%). The baseline characteristics of the future cases were similar to those of the current cases (supplementary table 8). Supplementary table 9 presents detailed information on characteristics for the hazard and reference periods.

**Table 1:** Baseline characteristics of eligible patients and current cases

Characteristics	Eligible patients (n=317 446)		Current cases (n=248 579)	
**Age (years), mean (SD)**	78.4 (0.01)		78.3 (0.02)	
**Male**	169 485 (53.4)		133 158 (53.6)	
**Comorbidities**				
Hypertension	197 349 (62.2)		155 411 (62.5)	
Heart failure	33 910 (11.3)		26 627 (10.7)	
Diabetes mellitus	102 673 (32.3)		81 330 (32.7)	
Dyslipidaemia	76 099 (24.0)		60 442 (24.3)	
Chronic kidney disease	63 408 (20.0)		49 676 (20.0)	
Chronic liver disease	21 356 (6.7)		16 997 (6.8)	
Asthma	22 249 (7.0)		17 661 (7.1)	
Chronic obstructive pulmonary disease	51 496 (16.2)		40 677 (16.4)	
Gastrointestinal ulcer or GERD	58 103 (18.3)		45 864 (18.5)	
Dementia	35 252 (11.1)		27 290 (11.0)	
Parkinson’s disease	12 097 (3.8)		9459 (3.8)	
Epilepsy	2748 (0.9)		2103 (0.9)	
Mental illness*	34 009 (10.7)		26 868 (10.8)	
Schizophrenia	1433 (0.5)		1145 (0.5)	
Osteoporosis	23 028 (7.3)		18 129 (7.3)	
Alcohol or drug abuse	420 (0.1)		352 (0.1)	
**Use of drugs with anticholinergic activity**				
Antihistamines	218 816 (68.9)		171 518 (69.0)	
Gastrointestinal antispasmodics	129 764 (40.9)		102 000 (41.0)	
Diuretics	107 398 (33.8)		84 003 (33.8)	
Bronchodilators	86 946 (27.4)		68 181 (27.4)	
Antiemetics or antivertigo agents	65 409 (20.6)		51 136 (20.6)	
Antipsychotics	56 576 (17.8)		44 122 (17.8)	
Antidepressants	35 718 (11.3)		27 874 (11.2)	
Antiepileptics	29 021 (9.1)		22 600 (9.1)	
Muscle relaxants	26 740 (8.4)		20 916 (8.4)	
Genitourinary antispasmodics	26 381 (8.3)		20 614 (8.3)	
Antiparkinson agents	17 377 (5.5)		13 550 (5.5)	
Antiarrhythmic drugs	277 (0.1)		219 (0.1)	

Values are numbers (%), unless indicated otherwise. Eligible patients were identified as those with a diagnosis of major cardiovascular event and current cases as eligible patients matched with future cases.

GERD=gastroesophageal reflux disease; SD=standard deviation.

* Mental illness included depression, bipolar disorder, and anxiety.

### Difference in total anticholinergic burden between periods

Among the patients with discordant levels of anticholinergic burden in different periods, more patients carried higher levels of anticholinergic burden during hazard periods than during reference periods. For example, 17 603 current cases had 1-2 points of anticholinergic burden in the hazard period with 0 points in the reference period, while 8507 current cases had the opposite anticholinergic burden. Among the future cases in the control crossover analysis, however, there were 14 247 and 10 436 future cases in the respective anticholinergic burden categories (table 2). The distribution of total anticholinergic burden in the hazard and reference periods indicated the average anticholinergic burden was higher in the hazard period among current cases, while this pattern was not observed among future cases (supplementary figs 4 and 5).

**Table 2:** Results of case-case-time-control analysis

Total anticholinergic burden score	Total	Higher burden in hazard period*	Higher burden in reference period	**Odds ratio (95% CI)**	
**1-2 *v* 0 points**					
Case crossover	26 110	17 603	8507	1.86 (1.83 to 1.90)	
Control crossover	24 683	14 247	10 436	1.35 (1.33 to 1.38)	
Case-case-time-control†	—	—	—	1.38 (1.34 to 1.42)	
**≥3 *v* 0 points**					
Case crossover	45 384	33 174	12 210	2.91 (2.86 to 2.96)	
Control crossover	36 368	21 363	15 005	1.43 (1.41 to 1.46)	
Case-case-time-control†	—	—	—	2.03 (1.98 to 2.09)	
**≥3 *v* 1-2 points**					
Case crossover	30 880	19 432	11 448	1.56 (1.53 to 1.59)	
Control crossover	24 627	12 703	11 924	1.06 (1.04 to 1.08)	
Case-case-time-control†	—	—	—	1.48 (1.44 to 1.52)	

Data are numbers of participants unless indicated otherwise. The case-case-time-control analysis incorporated two self-controlled analyses—a case crossover analysis and a control crossover analysis consisting of future cases to address confounding by indication and potential protopathic bias, respectively.

CI=confidence interval.

* Number of patients with higher burden in the hazard period for each corresponding anticholinergic burden category; for 1-2 *v* 0 group, patients with total burden of 1-2 points and 0 points in the reference period were counted; for ≥3 *v* 1-2 group, patients with total burden of ≥3 points and 1-2 points in the reference period were counted; and so on.

† The odds ratios of case-case-time-control analysis were obtained by dividing odds ratio of case crossover by odds ratio of control crossover.

### Association between recent anticholinergic burden and acute cardiovascular events

In the comparison of 1-2 versus 0 points of an anticholinergic burden, the odds ratio was 1.86 (95% confidence interval 1.83 to 1.90) in the case crossover analysis and 1.35 (1.33 to 1.38) in the control crossover analysis, which yielded a case-case-time-control odds ratio of 1.38 (1.34 to 1.42). Similar results were found in the comparison of ≥3 versus 0 points (2.03, 1.98 to 2.09) and ≥3 versus 1-2 points (1.48, 1.44 to 1.52; table 2).

When we analysed individual cardiovascular events, the results were generally consistent with the main analysis. The highest case-case-time-control odds ratio was observed in the cardiovascular death group in the comparison of ≥3 versus 0 points of anticholinergic burden (2.43, 2.25 to 2.63; table 3). Supplementary tables 10 and 11 present the frequencies of patients in different anticholinergic burden categories in the subgroup analysis.

**Table 3:** Summary of results of subgroup analysis and sensitivity analyses

Analyses	Total anticholinergic burden score			
	1-2 *v* 0 points	≥3 *v* 0 points	≥3 *v* 1-2 points	
Main analysis*	1.38 (1.34 to 1.42)	2.03 (1.98 to 2.09)	1.48 (1.44 to 1.52)	
Subgroup analysis				
Myocardial infarction	1.34 (1.24 to 1.45)	2.20 (2.05 to 2.37)	1.64 (1.52 to 1.77)	
Ischaemic stroke	1.42 (1.35 to 1.50)	2.05 (1.96 to 2.15)	1.44 (1.37 to 1.52)	
Haemorrhage stroke	1.26 (1.16 to 1.37)	1.62 (1.50 to 1.74)	1.28 (1.18 to 1.40)	
Arrhythmias	1.41 (1.33 to 1.49)	2.17 (2.06 to 2.28)	1.54 (1.46 to 1.63)	
Conduction disorder	1.29 (1.08 to 1.53)	1.82 (1.56 to 2.13)	1.42 (1.19 to 1.68)	
Syncope	1.19 (1.00 to 1.42)	1.84 (1.58 to 2.15)	1.55 (1.31 to 1.83)	
Cardiovascular death	1.43 (1.32 to 1.55)	2.43 (2.25 to 2.63)	1.70 (1.58 to 1.83)	
Sensitivity analysis				
Cut-off point for anticholinergic burden category†				
0, 1-4, ≥5	1.54 (1.51 to 1.58)	2.46 (2.38 to 2.53)	—	
0, 1-9, ≥10	1.70 (1.67 to 1.74)	2.90 (2.75 to 3.06)	—	
Length of hazard and reference periods 14 days	1.46 (1.43 to 1.50)	1.94 (1.89 to 1.99)	1.33 (1.28 to 1.37)	
Length of washout periods				
60 days	1.34 (1.31 to 1.38)	2.02 (1.98 to 2.07)	1.51 (1.47 to 1.55)	
120 days	1.30 (1.27 to 1.34)	1.97 (1.93 to 2.02)	1.51 (1.47 to 1.55)	
Length of interval between event dates of future cases and current cases 120-240 days‡	1.40 (1.36 to 1.45)	2.23 (2.16 to 2.30)	1.59 (1.54 to 1.64)	
Adjusted by drug dosing	2.81 (2.74 to 2.88)	5.43 (5.26 to 5.62)	1.93 (1.88 to 1.99)	
Adjusted by the duration of drug used				
Used >3 days within 30 days	1.74 (1.69 to 1.79)	2.15 (2.09 to 2.22)	1.24 (1.20 to 1.28)	
Used >7 days within 30 days	1.25 (1.21 to 1.28)	1.32 (1.27 to 1.36)	1.06 (1.02 to 1.10)	
Adjusted by time varying covariates	1.34 (1.31 to 1.38)	1.86 (1.81 to 1.91)	1.38 (1.34 to 1.42)	
Other scales for measuring burden				
ADS	1.40 (1.36 to 1.44)	2.20 (2.15 to 2.26)	1.57 (1.53 to 1.61)	
GABS	1.42 (1.38 to 1.46)	2.25 (2.19 to 2.31)	1.59 (1.54 to 1.63)	
m-ACB	1.36 (1.32 to 1.40)	2.07 (2.02 to 2.13)	1.52 (1.48 to 1.56)	
KABS	1.41 (1.37 to 1.45)	2.08 (2.03 to 2.13)	1.48 (1.43 to 1.52)	
Lag time inserted				
3 days	1.23 (1.20 to 1.27)	1.70 (1.66 to 1.75)	1.38 (1.34 to 1.42)	
7 days	1.14 (1.11 to 1.17)	1.44 (1.40 to 1.48)	1.26 (1.23 to 1.30)	
Exclusion of patients with cardiovascular drugs§	1.48 (1.40 to 1.55)	1.99 (1.91 to 2.06)	1.35 (1.28 to 1.42)	
Crude case crossover analysis (n=317 446)	1.83 (1.80 to 1.86)	2.93 (2.89 to 2.98)	1.60 (1.57 to 1.63)	
Case-time-control analysis (n=263 165)	1.59 (1.55 to 1.64)	2.46 (2.39 to 2.52)	1.54 (1.50 to 1.59)	
Restriction analysis by selecting patients with chronic conditions	1.34 (1.30 to 1.39)	2.02 (1.96 to 2.08)	1.51 (1.46 to 1.55)	

Values are odds ratios (95% confidence intervals).

ADS=Anticholinergic Drug Scale; GABS=German Anticholinergic Burden Scale; KABS=Korean Anticholinergic Activity Scale; m-ACB=Modified Anticholinergic Cognitive Burden Scale.

* Median time interval between event dates of current and future cases in main analysis 62 days; mean time interval 68.4 days.

† Corresponding fields are 1-4 *v* 0, ≥5 *v* 0, and ≥5 *v* 1-4; 1-9 *v* 0, ≥10 *v* 0; the comparisons of ≥5 *v* 1-4 and ≥10 *v* 1-9 were omitted because no additional information was required.

‡ Median time interval between event dates of current and future cases in sensitivity analysis 122 days; mean time interval 129.0 days.

§ Patient number after exclusion: current cases=114 838, future cases=116 223.

### Sensitivity analyses

The results from the sensitivity analyses were consistent with the primary analysis. Recently raised anticholinergic burden was associated with acute cardiovascular events after varying the cut-off point for groups, length of time windows, adjusting the anticholinergic burden by drug dosing or duration, and potential time varying confounders. Specifically, when we changed the definition of anticholinergic burden categories from ≥3 points to ≥5 and ≥10 points, the odds ratios increased and a greater difference in anticholinergic burden was found between the hazard and reference periods. The odds ratios were 2.46 (95% confidence interval 2.38 to 2.53) and 2.90 (2.75 to 3.06) in comparisons of ≥5 versus 0 points and ≥10 versus 0 points, respectively, compared with an odds ratio of 2.03 (1.98 to 2.09) for ≥3 versus 0 points (table 3). We used four additional scales for anticholinergic burden measurement to investigate the association and the results were similar to the use of the Anticholinergic Cognitive Burden Scale (table 3, supplementary table 12).

When we inserted a lag time of three or seven days before the index date, the results remained significant, but we observed lower odds ratios. For example, in the comparison of ≥3 versus 0 points, the odds ratios were 1.70 (1.66 to 1.75) and 1.44 (1.40 to 1.48) for a three day and seven day lag time, respectively, lower than the odds ratio of 2.03 (1.98 to 2.09) in the primary analysis (table 3). The analysis that excluded patients using cardiovascular drugs in the previous year included 114 838 current cases and 116 223 future cases, and yielded odds ratios of 1.48 (1.40 to 1.55), 1.99 (1.91 to 2.06), and 1.35 (1.28 to 1.42) for comparisons of 1-2 versus 0 points, ≥3 versus 0 points, and ≥3 versus 1-2 points, respectively. The results of other sensitivity analyses after excluding patients with special conditions in the baseline period were consistent with the main analysis, as presented in supplementary table 13.

Using the case-time-control design for time trends adjustment, the results were comparable to the case-case-time-control analysis, with case-time-control odds ratios of 1.59 (1.55 to 1.64), 2.46 (2.39 to 2.52), and 1.54 (1.50 to 1.59) for the comparisons of 1-2 versus 0 points, ≥3 versus 0 points, and ≥3 versus 1-2 points, respectively (table 3, supplementary tables 14 and 15). Similarly, we found a greater difference in anticholinergic burden between the hazard and reference periods among cases but not among external controls without acute cardiovascular events (supplementary figs 6-8). The results were consistent when the disease risk scores were determined based on the covariates within one year before the reference period.

In the restrictive analysis that included only patients with chronic conditions, the results were consistent with the main analysis, with odds ratios of 1.34 (1.30 to 1.39), 2.02 (1.96 to 2.08), and 1.51 (.46 to 1.55) for comparisons of 1-2 versus 0 points, ≥3 versus 0 points, and ≥3 versus 1-2 points, respectively (table 3).

## Discussion

### Principal findings

In this nationwide, population based study, we found that older adults with acute cardiovascular events had a higher total anticholinergic burden in the 30 day hazard windows compared with the reference windows, indicating an association between recently raised anticholinergic burden and acute cardiovascular events. We found that although the results were substantially diminished after considering the effects of protopathic bias through the case-case-time-control design, the bias did not fully explain the increased risk. Furthermore, the association was strengthened by the evidence of a dose-response relation between anticholinergic burden and risk of acute cardiovascular events. We obtained consistent results throughout a series of sensitivity analyses based on various scenarios and different anticholinergic burden scales.

### Strengths and weaknesses of this study

This study evaluates the association between recent anticholinergic burden and risk of acute cardiovascular events. We considered variation in total anticholinergic burden in different periods before the events by including all systemic drugs for anticholinergic burden calculation, even if the drugs were only for short term use. The case-case-time-control analysis enabled us to eliminate confounding by indication owing to the self-controlled design. Our study revealed a substantial difference in the results obtained through case-case-time-control analysis compared with case-time-control analysis, highlighting the issues of protopathic bias encountered in previous studies.^12^ ^13^ ^14^ ^16^ ^28^ However, the case-case-time-control analysis and the sensitivity analysis with different lag times addressed these concerns and revealed the robustness of the association. These findings suggest a strong association between recently raised anticholinergic burden and an increased risk of acute cardiovascular events.

Our study had some limitations. The database only recorded the use of drugs reimbursed by the National Health Insurance programme in Taiwan. We did not include over-the-counter drugs or those paid for by patients, which could have resulted in a misclassification of anticholinergic burden categories. However, the results from a restrictive analysis that only selected patients with chronic diseases suggested that the effects of this issue were minor. Additionally, there was no specific cut-off point for anticholinergic burden to differentiate the associated risks of acute cardiovascular events. We used a threshold of ≥3 points to identify those with a high anticholinergic burden and conducted sensitivity analyses by redefining the threshold to ≥5 and ≥10. The results were consistent across all thresholds. 

While the self-controlled analysis inherently eliminated time constant confounders, and we further included certain time varying confounders in the regression models for adjustment, it is important to acknowledge the potential for residual confounding owing to time varying indications. Furthermore, there was no consensus on a standard scale to measure anticholinergic burden. Different scales might have slight differences in drugs covered, which could be better suited for different countries and healthcare systems. For example, the German Anticholinergic Burden Scale has been updated to include more recently launched drugs, and the Korean Anticholinergic Activity Scale covers more drugs that are used in Asian countries. Nevertheless, we obtained consistent results across the anticholinergic burden scales mainly because the drugs that were frequently used by study participants were covered by most scales. Finally, although the claims database provided complete records of prescriptions, the lack of information on drug adherence prevented us from confirming whether the patients actually took their drugs, leading to possible misclassification bias. However, this bias did not affect our conclusion because we could expect the effects of drug adherence to be consistent across the hazard and reference windows, resulting in a similar impact on estimates.

### Strengths and weaknesses in relation to other studies

Previous research has reported an association between anticholinergic burden and cardiovascular diseases. However, while these studies evaluate long term outcomes, they only classify patients based on their baseline anticholinergic burden, without considering changes in anticholinergic burden over time. Furthermore, these studies could be subject to protopathic bias because they do not consider that drugs with anticholinergic effects, such as antihistamines and gastrointestinal drugs, might be used to alleviate early symptoms of cardiovascular events.^10^ ^12^ ^13^ ^14^ Our study, which employed case-case-time-control and case-time-control analyses, revealed that protopathic bias could be substantial.

### Meaning of the study: possible explanations and implications

Our findings were supported by biological plausibility from existing evidence. Drugs with anticholinergic activity have pro-arrhythmic and pro-ischaemic effects.^15^ The parasympathetic nervous system plays an important role in the regulation of cardiac automaticity and contractility, and the inhibition of parasympathetic control over the cardiovascular system could increase haemodynamic lability.^46^ ^47^ This effect might lead to tachyarrhythmias and increased oxygen requirements, which are associated with a higher risk of ischaemic events such as myocardial infarction, ischaemic strokes, and even sudden cardiac death. Furthermore, cardiac ischaemic events can cause cardiac dysrhythmias.^15^ ^48^ Other mechanisms, such as automatic imbalance and inflammatory responses related to anticholinergic effects, have been proposed in addition to the pro-arrhythmic and pro-ischaemic effects.^49^ ^50^ ^51^ ^52^ Additionally, some studies have indicated that the onset of anticholinergic effects can be rapid after using certain drugs.^47^ ^53^ Biological plausibility supports that recently raised anticholinergic burden could be associated with adverse events.

Protopathic bias might arise from the increased use of drugs to manage early symptoms of cardiovascular events before diagnosis. Determining whether the anticholinergic burden caused the events or if the events led to increased drug use is challenging. To minimise possible protopathic bias, we conducted control analyses using future cases, which provided estimates on trends in drug use that might be the source of protopathic bias. The trends were then further adjusted for in the case-case-time-control analysis (supplementary information 2).^16^ ^28^ ^54^ ^55^ We also obtained a lower risk estimate when we manipulated the lag time to investigate the drugs in the sensitivity analyses. These findings highlighted the need to consider protopathic bias when interpreting the results from observational studies on this topic.

This study identified an association between a recently raised anticholinergic burden and an increased risk of acute cardiovascular events in older adults. The dose-response relation between anticholinergic burden and the risk of acute cardiovascular events supported probable causation. The study does not infer that a specific drug could cause cardiovascular events, but rather that a cumulative anticholinergic effect might be related to the increased risk. These findings highlight the need for healthcare providers to reduce unnecessary drug use and closely monitor patients for potential adverse cardiovascular effects. Given that many drugs have anticholinergic effects in addition to their primary mechanism of action, establishing an automated system to assist in the detection of anticholinergic burden could be helpful.

### Unanswered questions and future research

Although our study provides findings on an association between raised anticholinergic burden and risk of acute cardiovascular events, further research is needed to determine the threshold of tolerable anticholinergic burden and appropriate time intervals for monitoring to provide more practical recommendations for clinical application. Our study could provide a foundation for the development and inclusion of an anticholinergic index calculator in electronic health records to alert doctors when prescribing a new drug that contributes to the anticholinergic burden. Doctors might consider alternative drugs within the same therapeutic class that possess less anticholinergic effect, especially when patients are at an increased risk of cardiovascular events. Additionally, our study showed the potential for substantial protopathic bias, highlighting the need for future studies to address this methodological concern.

### Conclusions

Our study found an association between recently raised anticholinergic burden and an increased risk of acute cardiovascular events, even after addressing the issue of protopathic bias. We also showed a dose-response relation between anticholinergic burden and the risk of acute cardiovascular events, indicating probable causation. The findings were robust throughout a series of analyses that controlled for protopathic bias and confounders unless otherwise indicated by the presence of residual confounding. Given that many drugs have anticholinergic effects in addition to their primary mechanism of action, healthcare providers should increase their awareness of these effects and consider reducing unnecessary drug use.

**What is already known on this topic**

Previous studies have reported an association between anticholinergic burden and increased cardiovascular risk, but have not considered the potential issue of protopathic bias
Research has investigated the relation between anticholinergic burden and long term cardiovascular events, but variations in anticholinergic burden over time require investigation
Studies examining the effect of a recently raised anticholinergic burden on the risk of cardiovascular events are lacking

**What this study adds**

A recently raised anticholinergic burden was associated with an increased risk of acute cardiovascular events, even after addressing the issue of protopathic bias
A dose-response relation was found between anticholinergic burden and risk of acute cardiovascular events
The findings highlight the need to consider protopathic bias when interpreting the results from observational studies

## Data Availability

The authors remotely accessed the data from the data centre of the Ministry of Health and Welfare in Taiwan. Researchers interested in accessing this dataset could submit a formal application to the Taiwan Ministry of Health and Welfare to request access (No 488, Sect. 6, Zhongxiao E Rd, Nangang District, Taipei 115, Taiwan; website: https://dep.mohw.gov.tw/DOS/cp-2516-59203-113.html). No additional data available.

## Web extra

Extra material supplied by authors

Web appendix: Data supplement

## Notes

Contributors: WCH and ECCL initiated the collaborative project, developed the research question, designed the study protocol, and drafted the manuscript. WCH and ECCL are the guarantors. WCH, ASHY, DHTT, SCS, SJL, and ECCL edited the study protocol, interpreted the results, and reviewed the manuscript. ASHY wrote the statistical analysis plan and conducted the statistical analysis. The corresponding author attests that all listed authors meet authorship criteria and that no others meeting the criteria have been omitted.

The lead author (the manuscript’s guarantor) affirms that the manuscript is an honest, accurate, and transparent account of the study being reported; that no important aspects of the study have been omitted; and that any discrepancies from the study as planned (and, if relevant, registered) have been explained.

Dissemination to participants and related patient and public communities: The authors plan to submit the accepted manuscript to the National Cheng Kung University (NCKU) Hospital, NCKU College of Medicine, and Taiwan Food and Drug Administration (TFDA) to apply for publication on their press releases. They will also write a post on the official website of NCKU School of Pharmacy, which will tag all members in the research groups on Facebook and Twitter.

Provenance and peer review: Not commissioned; externally peer reviewed.
