# Supplementary material for: Association between recently raised anticholinergic burden and risk of acute cardiovascular events: nationwide case-case-time-control study
Source: BMJ. 2023 Sep 27;382:e076045. doi: 10.1136/bmj-2023-076045 (PMC10523277; doi:10.1136/bmj-2023-076045)
Supplement: Supplementary file 1 — Web appendix: Data supplement [file huaw076045.ww.pdf]

DATA SUPPLEMENT

- **Supplementary information 1.** Formula for calculating confidence intervals of the OR_{CCTC}.
- **Supplementary information 2.** Detailed information of the case-case-time-control (CCTC) design and case- time-control (CTC).
- **Supplementary table 1.** ICD-9-CM and ICD-10-CM codes for identification of acute cardiovascular events
- **Supplementary table 2.** Anticholinergic agents listed and scored in the ADS, ACB, GABS, m-ACB, and KABS scale (only listing medications available in Taiwan's NHIRD)
- **Supplementary table 3.** Definition of anticholinergic burden categories for anticholinergic burden in the hazard and reference period.
- **Supplementary table 4.** Comorbidities collected for baseline characteristics and used for disease risk score matching.
- **Supplementary table 5.** Medications with anticholinergic activity collected for baseline characteristics.
- **Supplementary table 6.** Covariates included for time-varying confounders adjustment in sensitivity analysis.
- **Supplementary table 7.** Different scales for anticholinergic burden measurement in sensitivity analysis.
- **Supplementary table 8.** Characteristics in baseline periods of current cases and future cases in the main analysis
- **Supplementary table 9.** Characteristics in hazard and reference periods of current cases and future cases in the main analysis
- **Supplementary table 10.** Results of subgroup analyses matching current cases and future cases on diagnoses of individual cardiovascular events.
- **Supplementary table 11.** Frequencies of participants in different anticholinergic burden categories in subgroup analysis.

- **Supplementary table 12.** Results of primary analysis and sensitivity analyses using different scales for anticholinergic burden measurement.
- **Supplementary table 13.** Results of sensitivity analyses excluding patients with special conditions in baseline period.
- **Supplementary table 14.** Results of primary analysis and sensitivity analyses of the crude case-crossover and case-time-control analysis.
- **Supplementary table 15.** Frequencies of participants in different anticholinergic burden categories in primary analysis and sensitivity analyses of the crude case-crossover and case-time-control analysis.
- **Supplementary figure 1.** Illustration for study design of the crude case-crossover analysis.
- **Supplementary figure 2.** Illustrations for study design of the case-time-control (CTC) analysis.
- **Supplementary figure 3.** Flowchart for selection of cases and external controls in the case-time-control (CTC) analysis.
- **Supplementary figure 4.** Distribution of total anticholinergic burden in hazard and reference periods among current cases and future cases in the case-case-time-control analysis.
- **Supplementary figure 5.** Box and whisker plot for total anticholinergic burden in hazard and reference periods among current cases and future cases in the case-case-time-control analysis.
- **Supplementary figure 6.** Distribution and box and whisker plot for total anticholinergic burden in hazard and reference periods among eligible patients in the crude case-crossover analysis.
- **Supplementary figure 7.** Distribution of total anticholinergic burden in hazard and reference periods among cases and external controls in the case-time-control analysis.
- **Supplementary figure 8.** Box and whisker plot for total anticholinergic burden in hazard and reference periods among cases and external controls in the case-time-control analysis.

Supplementary information 1. Formula for calculating confidence intervals of the $\mathbf{OR_{CCTC}}$.

The confidence intervals were calculated using the conditional logistic regression model, based on the simplified formula provided below.

$$\log\left(\frac{\Pr(Y=1|X)}{1-\Pr(Y=1|X)}\right) = \beta_0 + \beta_1 X + \cdots\ ,\ \Pr(Y=1|X)\ as\ probability\ of\ event$$

$$\text{The odds ratio between Y and X}:\ OR_{xy} = \exp(\beta_1)$$

$$\text{The } (1-\alpha)\% \text{ two}-\text{sided confidence interval for } \beta_1:\ \hat{\beta}_1 \pm z_{1-\frac{\alpha}{2}} s_{\hat{\beta}_1}\ ,\ s_{\hat{\beta}_1} \text{as standard error of } \hat{\beta}_1$$

$$\text{The } (1-\alpha)\% \text{ two}-\text{sided confidence interval for } OR_{xy}:\ (OR_{LL}, OR_{UL}) = \exp\left(\hat{\beta}_1 \pm z_{1-\frac{\alpha}{2}} s_{\hat{\beta}_1}\right)$$

In CCTC analysis, we have OR_{case} and $OR_{control}$, here we use OR_A (OR_{ALL}, OR_{AUL}) and OR_B (OR_{BLL}, OR_{BUL})

$$OR_{case} = OR_A = \exp(\beta_{A1})$$

$$OR_{control} = OR_B = \exp(\beta_{B1})$$

$$(OR_{ALL}, OR_{AUL}) = \exp\left(\hat{\beta}_{A1} \pm z_{1-\frac{\alpha}{2}} s_{\hat{\beta}_{A1}}\right) \Rightarrow s_{\hat{\beta}_{A1}} = \frac{\ln(OR_{AUL}) - \ln(OR_{ALL})}{2z_{1-\frac{\alpha}{2}}}$$

$$(OR_{BLL}, OR_{BUL}) = \exp\left(\hat{\beta}_{B1} \pm z_{1-\frac{\alpha}{2}} s_{\hat{\beta}_{B1}}\right) \Rightarrow s_{\hat{\beta}_{B1}} = \frac{\ln(OR_{BUL}) - \ln(OR_{BLL})}{2z_{1-\frac{\alpha}{2}}}$$

$$OR_{CCTC} = \frac{OR_{case}}{OR_{control}} = \frac{OR_A}{OR_B} = \frac{\exp(\beta_{A1})}{\exp(\beta_{B1})}\ \text{, here we use } OR_C\ (OR_{CLL}, OR_{CUL})$$

$$\beta_{c1} = \ln(OR_C) = \ln\left(\frac{OR_A}{OR_B}\right),\ s_{\hat{\beta}_{C1}} = \sqrt{{s_{\hat{\beta}_{A1}}}^2 + {s_{\hat{\beta}_{B1}}}^2}$$

$$(OR_{CLL}, OR_{CUL}) = \exp\left(\beta_{c1} \pm z_{1-\frac{\alpha}{2}} s_{\hat{\beta}_{C1}}\right) = \exp\left[\ln\left(\frac{OR_A}{OR_B}\right) \pm z_{1-\frac{\alpha}{2}}\sqrt{{s_{\hat{\beta}_{A1}}}^2 + {s_{\hat{\beta}_{B1}}}^2}\right]$$

$$= \exp\left[\ln\left(\frac{OR_A}{OR_B}\right) \pm z_{1-\frac{\alpha}{2}}\sqrt{\left(\frac{\ln(OR_{AUL}) - \ln(OR_{ALL})}{2z_{1-\frac{\alpha}{2}}}\right)^2 + \left(\frac{\ln(OR_{BUL}) - \ln(OR_{BLL})}{2z_{1-\frac{\alpha}{2}}}\right)^2}\right]$$

Supplementary information 2. Detailed information of the case-case-time-control (CCTC) design and case- time-control (CTC).

Both the case-time-control (CTC) and the case-case-time-control (CCTC) designs assume that the observed odds ratio among cases is the product of the odds ratio for the causal effect of the exposure on outcome multiplied by the odds ratio for exposure trends over calendar time:

$$OR_{case} = OR_{causal} \times OR_{time\text{-}trend}$$

By contrast, the odds ratio among the controls can only be explained by exposure trends without specific causality:

$$OR_{control} = OR_{time\text{-}trend}$$

To obtain the odds ratio for the causality of exposure on outcome (OR_{causal}), the cases' odds ratio ($OR_{causal} \times OR_{time\text{-}trend}$) is divided by the corresponding odds ratio of concurrent matched controls ($OR_{time\text{-}trend}$).

The main difference between the two control analyses is that the CCTC design uses future cases as the control group, whereas the CTC design employs external non-case controls in which the events did not occur. The CCTC design assumes that the trend in exposure over calendar time is the same for current and future cases because both of them experience the events (1). If there is an issue of protopathic bias from the medications used for the prodromes of the events, the exposure time trends will be observed in both groups. This allows the reference time periods sampled from future cases in CCTC analysis to provide a better estimate of the time effect, when compared to the periods sampled from external non-case controls in the CTC design. The figures illustrate the concepts in the case-time-control and case-case-time-control design and how the future case methods can address the protopathic bias under discussion.

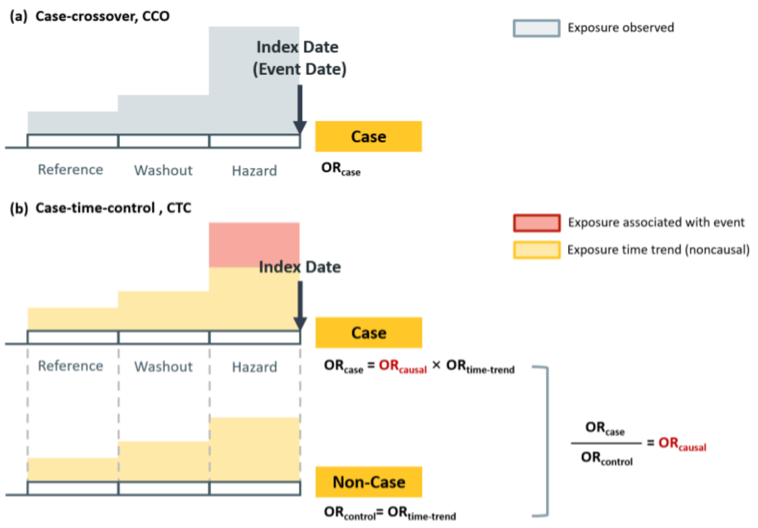

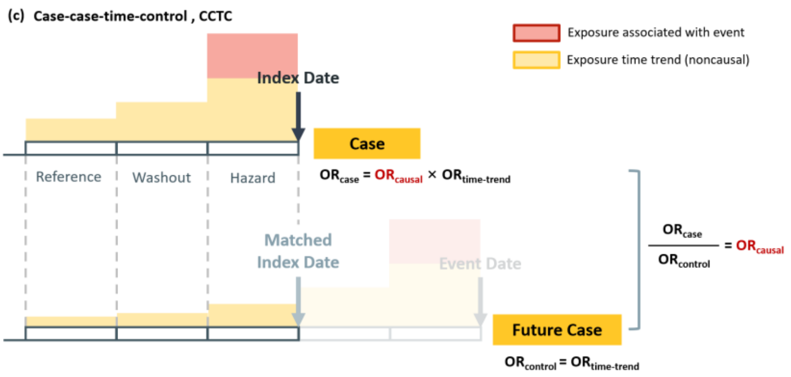

Illustration of the concepts in the case-time-control and case-case-time-control design

(a) The case-crossover analysis. (b) The assumptions of case-time-control design. (c) The assumptions of case-case-time-control design.

**Note:* These figures are intended for simple illustration of the study designs only.

Reference:

1. Wang S, Linkletter C, Maclure M, Dore D, Mor V, Buka S, et al. Future cases as present controls to adjust for exposure trend bias in case-only studies. *Epidemiology*. 2011;22(4):568-74.

Supplementary table 1. ICD-9-CM and ICD-10-CM codes for identification of acute cardiovascular events

Acute CV events	ICD-9-CM	ICD-10-CM
Myocardial infarction	410	I21
Other acute and subacute forms of ischaemic heart disease	411	I22-I24
Stroke		
Heamorrhagic stroke	430-432	I60-I62
Ischaemic stroke	433, 434	I63
Acute cerebrovascular disease, undefined	436	I67.81
Conduction disorders	426	I44, I45
Cardiac dysrhythmias	427	I46-I49
Syncope	780.2	R55
Cardiovascular mortality*†	401-405, 410-414, 415-417, 426-428, 430-437, 440-441, 443	I10-I16, I20-I25, I26-I28, I44-I50, I60-I63, I67-I69, I70-I71, I73

* Using the primary diagnosis for the cause of death in National Register of Deaths

† Defined as deaths attributable to ischaemic heart diseases, strokes, hypertension, pulmonary heart diseases, heart failure, conduction disorders, dysrhythmias, or artery disease including atherosclerosis, aortic aneurysm and dissection

‡ A 3-digit ICD code includes more specific fourth and fifth digits. For example, the ICD-9-CM code "410" includes the wildcard codes "410.xx".

Supplementary table 2. Anticholinergic agents listed and scored in the ADS, ACB, GABS, m-ACB, and KABS scale (only listing medications available in Taiwan's NHIRD)

Medication	ATC code	ADS	ACB	GABS	m-ACB	KABS
**Analgaesics**						
Etoricoxib	M01AH05	.	.	1	.	.
Fentanyl	N01AH01, N02AB03	1	1	1	1	1
Meperidine / pethidine	N02AB02	2	2	2	2	2
Morphine	N02AA01	1	1	1	1	1
Nefopam	N02BG06	.	2	.	2	2
Oxycodone	N02AA05	1	.	1	.	1
Tramadol	N02AJ13, N02AX02	1	.	2	.	2
**Antidepressants**						
Amitriptyline	N06AA09	3	3	3	3	3
Bupropion	N06AX12	0	1	1	1	1
Citalopram	N06AB04	0	.	1	1	1
Clomipramine	N06AA04	3	3	3	3	3
Dothiepin	N06AA16	.	.	.	3	.
Doxepin	N06AA12	3	3	3	3	3
Duloxetine	N06AX21	0	.	.	.	0
Escitalopram	N06AB10	0	.	1	1	1
Fluoxetine	N06AB03	1	.	1	.	1
Fluvoxamine	N06AB08	1	1	1	1	1

Imipramine	N06AA02	3	3	3	3	3
Maprotiline	N06AA21	.	.	2	2	.
Mirtazapine	N06AX11	0	.	1	1	1
Paroxetine	N06AB05	1	3	2	3	2
Sertraline	N06AB06	1	.	1	.	0
Trazodone	N06AX05	0	1	1	1	1
Venlafaxine	N06AX16	0	1	1	1	1
**Antiepileptics**						
Carbamazepine	N03AF01	2	2	2	2	1
Clonazepam	N03AE01	1	.	1	.	1
Oxcarbazepine	N03AF02	2	2	2	2	2
Valproic acid	N03AG01	1	.	1	.	0
**Antigout agents**						
Colchicine	M04AC01, M04A	0	1	.	1	0
**Antihistamines**						
Alimemazine	R06AD01	.	1	.	1	1
Brompheniramine	R06AB01	3	3	.	3	3
Buclizine	R06AE01, R06AE51	.	.	.	3	.
Carbinoxamine	R06AA08	3	3	.	3	3
Cetirizine	R06AE07	0	1	1	1	1
Chlorpheniramine	R06AB04, B05XC, R05CA10, R05DB, R06AB54	3	3	3	3	3

Clemastine	R06AA04	3	3	3	3	3
Cyproheptadine	R06AX02	2	2	3	2	2
Desloratadine	R06AX27	.	1	1	1	1
Dexchlorpheniramine	R06AB02	.	.	.	3	3
Dimenhydrinate	R06AA02	3	3	3	3	3
Diphenhydramine	R06AA02, C05AX, N02CA52, R01AB02, R01AX30	3	3	3	3	3
Diphenylpyraline‡	R05X‡	.	.	.	3	3
Doxylamine	R03CB53, R05CB10, R06AA	.	3	1	3	3
Fexofenadine	R06AX26	0	.	1	.	0
Homochlorcyclizine	R06AE91	.	.	.	3	3
Hydroxyzine	N05BB01	3	3	3	3	3
Ketotifen	R06AX17	1	.	.	1	1
Levocetirizine	R06AE09	.	1	1	1	1
Loratadine	R06AX13	0	1	1	1	1
Meclizine	R06AE05	3	3	.	3	3
Mequitazine	R06AD07	.	.	.	3	3
Phenindamine	R06AX04			.	2	.
Piprinhydrinate	R06AA07	.	.	.	3	3
Promethazine	R06AD02	3	3	1	3	.
Triprolidine‡	R01BA52‡	.	.	.	2	2

**Antiinfectives**						
Cycloserine	J04AB01	1	.	.	.	0
Ampicillin	J01CA01, J01CR50, J01CR01	1	.	1	.	0
Cefamandole	J01DC03	1	.	.	.	0
Cefoxitin	J01DC01	1	.	.	.	0
Clindamycin	J01FF01	1	.	1	.	0
Gentamicin	J01GB03	1	.	1	.	0
Piperacillin	J01CA12, J01CR05	1	.	1	.	0
Vancomycin	J01XA01	1	.	1	.	0
**Antiparkinson agents**						
Amantadine	N04BB01	1	2	2	2	2
Benztropine	N04AC01	3	3	.	3	3
Biperiden	N04AA02	.	.	.	3	3
Carbidopa‡	N04BA02‡, N04BA03‡	0	.	.	.	0
Entacapone	N04BX02	0	.	1	.	.
Levodopa‡	N04BA02‡, N04BA03‡	0	.	1	.	0
Pramipexole	N04BC05	0	.	1	.	0
Rasagiline	N04BD02	.	.	.	1	.
Rotigotine	N04BC09	.	.	1	.	.
Selegiline	N04BD01	0	.	1	.	0
Trihexyphenidyl / benzhexol	N04AA01	3	3	3	3	3

Antipsychotics						
Amisulpride	N05AL05	.	.	.	1	1
Aripiprazole	N05AX12	.	1	1	1	1
Chlorpromazine	N05AA01	3	3	.	3	3
Chlorprothixene	N05AF03	.	.	.	3	3
Clozapine	N05AH02	3	3	3	3	3
Flupentixol	N05AF01, N06CA02	.	.	.	1	1
Fluphenazine	N05AB02	1	.	1	.	.
Haloperidol	N05AD01	0	1	2	1	1
Levomepromazine / methotrimeprazine	N05AA02	2	2	3	2	2
Lithium	N05AN01	0	.	1	.	0
Loxapine	N05AH01	2	2	2	2	2
Olanzapine	N05AH03	1	3	2	3	3
Paliperidone	N05AX13	.	1	1	1	1
Perphenazine	N05AB03	1	3	1	3	2
Pimozide	N05AG02	2	2	2	2	2
Prochlorperazine	N05AB04	1	.	.	1	.
Quetiapine	N05AH04	0	3	2	3	2
Risperidone	N05AX08	0	1	1	1	1
Sulpiride	N05AL01	.	.	.	1	.
Thioridazine	N05AC02	3	3	3	3	3
Trifluoperazine	N05AB06	1	3	.	3	.

Ziprasidone	N05AE04	.	.	1	1	1
Zotepine	N05AX11	.	.	.	2	2
Zuclopenthixol	N05AF05	.	.	.	2	2
**GI antispasmodics**						
Alverine	A03AX08	.	1	.	1	0
Atropine	A03BA01, N03AA	3	3	3	3	3
Belladonna	A03CB02, N02CA72	.	2	.	2	3
Butinolin‡	A02AG‡	.	.	.	3	.
Butropium	A03AB91	.	.	.	3	.
Butylscopolamine	A03BB01	.	.	.	3	.
Clidinium‡	A03CA02‡	.	1	.	1	2
Dicyclomine	A03AA07, A03CC	3	3	.	3	3
Hyoscine	A03BB01	3	3	.	.	.
Hyoscyamine	A03BA03, A03CB31	3	3	.	.	3
Mebeverine	A03AA04	.	.	.	1	1
Otilonium bromide	A03AB06	.	.	.	3	3
Oxapium	A03AB	.	.	.	3	3
Oxyphencyclimine	A03AA01	.	.	.	3	.
Pipethanate	A03BA92	.	.	.	3	.
Propantheline	A03AB05	3	3	.	3	.
Scopolamine	A03BB01, R06AE55	3	3	3	3	3
Scopolia	A07AX, A03EA	.	3	.	3	3

Timepidium	A03AB19	.	.	.	3	3
Trimebutine	A03AA05	.	.	.	1	1
Valethamate bromide	A03AB93	.	3	.	3	3
**Other GI agents**						
Cimetidine	A02BA01	2	1	2	1	2
Domperidone	A03FA03	.	.	1	.	0
Famotidine	A02BA03	1	.	1	.	0
Loperamide	A07DA03	1	1	2	1	1
Metoclopramide	A03FA01	0	.	1	.	0
Nizatidine	A02BA04	1	.	.	.	0
Pirenzepine	A02BX03	.	.	.	3	.
Ranitidine	A02BA02	2	1	2	1	1
Trospium	A03AB20	.	3	3	3	3
**Antiemetics / Antivertigo agents**						
Cinnarizine	N07CA02	.	.	.	1	1
Diphenidol	N07CA	.	.	.	2	2
**Benzodiazepines**						
Alprazolam	N05BA12	1	1	1	1	1
Chlordiazepoxide	N05BA02	1	.	1	.	1
Diazepam	N05BA01	1	1	1	1	1
Estazolam	N05CD04	1	.	.	1	1
Flunitrazepam	N05CD03	.	.	1	1	1

Flurazepam	N05CD01	1	.	1	.	1
Lorazepam	N05BA06	1	.	1	.	1
Midazolam	N05CD08	1	.	1	.	1
Oxazepam	N05BA04	1	.	1	1	.
Triazolam	N05CD05	1	.	1	.	1
**Cardiovascular drugs**						
Amiodarone	C01BD01	0	.	.	.	0
Atenolol	C07AB03	0	1	1	1	0
Captopril	C09AA01	0	.	.	.	0
Chlorthalidone	C09DA09	0	1	1	1	0
Digoxin	C01AA05	1	1	1	1	1
Diltiazem	C08DB01	1	.	1	.	0
Dipyridamole	B01AC07	1	1	1	1	0
Disopyramide	C01BA03	2	1	.	1	.
Doxazosin	C02CA04	.	.	.	1	0
Furosemide	C03CA01	1	1	1	1	1
Hydralazine	C02DB02	1	1	1	1	1
Hydrochlorothiazide	C03AA03, C09BA02, C09DA01, C09DA03, C09DA04, C09DA06, C09DA07, C09DA08, C09DX01, C09DX03, C09XA52	.	1	.	1	0
Isosorbide	B05BC	1	1	.	1	0
Isosorbide dinitrate	C01DA08	1	.	1	.	.

Isosorbide mononitrate	C01DA14	1	.	1	.	.
Metoprolol	C07AB02	0	1	1	1	0
Nifedipine	C08CA05	1	1	1	1	0
Quinidine	C01BA01	0	1	1	1	.
Triamterene‡	C03EA01‡	1	1	1	1	0
Warfarin	B01AA03	1	1	1	1	0
**Corticosteroids**						
Cortisone	H02AB10	1	.	.	.	0
Dexamethasone	H02AB02	1	.	1	.	0
Hydrocortisone	H02AB09	1	1	1	1	1
Methylprednisolone	H02AB04	1	.	1	.	0
Prednisolone	H02AB06, H02BX	1	1	1	1	1
Triamcinolone	H02AB08	1	.	1	.	0
**Immunosuppressants**						
Azathioprine	L04AX01	1	.	.	.	0
Cyclosporine	L04AD01	1	.	1	.	0
**Muscle relaxants**						
Baclofen	M03BX01	0	1	1	1	1
Carisoprodol	M03BA02, M03BA52	0	.	.	.	.
Cyclobenzaprine	M03BX08	2	2	.	2	2
Methocarbamol	M03BA03	.	.	1	3	1
Orphenadrine	M03BC01, M03BC51	3	3	3	3	3

Pridinol	M03BX03	.	.	.	3	3
Tizanidine	M03BX02	.	.	3	2	2
**Respiratory tract agents**						
Cloperastine‡	R05FB02‡	.	.	.	2	2
Codeine	R05DA04	1	1	1	1	1
Fluticasone-salmeterol	R03AK06	1	.	.	.	.
Glycopyrrolate	R03BB06	.	.	1	3	2
Glycopyrronium	R03AL04, R03AL07, R03AL09	.	.	1	.	.
Ipratropium	R03AL01	0	.	1	.	.
Pseudoephedrine	R03BB01, R03AL02, R03AL01	0	.	1	.	0
Theophylline	R03DA04, R03DA54, R03DB04	1	1	2	1	1
Tiotropium	R03BB04, R03AL06	.	.	1	.	.
**Genitourinary antispasmodics**						
Bromocriptine	G02CB01	1	.	1	.	0
Flavoxate	G04BD02	3	3	3	3	3
Oxybutynin	G04BD04	3	3	3	3	3
Pipoxolan	G04BD	.	.	.	3	0
Propiverine	G04BD06	.	3	3	3	3
Solifenacin	G04BD08	.	3	3	3	3
Tolterodine	G04BD07	3	3	3	3	3
Trospium	G04BD09	.	3	3	3	3

**Other combination drugs (containing anticholinergic agents) †‡**						
Butinolin, Butylscopolamine, Dicyclomine, Hyoscine, Pipethanate, Scopolamine, Chlordiazepoxide	A02AG	3	3	3	3	3
Propantheline, Scopolia	A02AX	3	3	.	3	3
Clidinium, Chlordiazepoxide	A03CA02	1	1	1	1	2
Atropine, Butylscopolamine, Hyoscine, Hyoscyamine, Scopolamine	A03CB	3	3	3	3	3
Belladonna, Scopolia	A09AA	.	3	.	3	3
Hydralazine, Hydrochlorothiazide	C02LA51	.	3	.	3	3
Hydrochlorothiazide, Triamterene	C03EA01	.	3	.	3	3
Atenolol, Chlorthalidone	C07CB03	1	1	1	1	0
Chlorpheniramine, Codeine	N02BE51	3	3	3	3	3
Carbidopa, Levodopa	N04BA02	0	.	1	.	0
Entacapone, Levodopa	N04BA03	0	.	1	.	0
Brompheniramine, Chlorpheniramine, Chlorpheniramine, Belladonna, Pseudoephedrine	R01BA51	3	3	3	3	3
Carbinoxamine, Chlorpheniramine, Fexofenadine, Loratadine, Triprolidine, Pseudoephedrine	R01BA52	3	3	3	3	3
Brompheniramine, Pseudoephedrine	R01BA53	3	3	1	3	3
Chlorpheniramine, Dimenhydrinate, Scopolamine, Theophylline	R03DA51	3	3	1	3	3
Chlorpheniramine, Theophylline	R03DA71	3	3	3	3	3

Chlorpheniramine, Diphenhydramine, Theophylline	R03DB01	3	3	3	3	3
Carbinoxamine, Chlorpheniramine, Dexchlorpheniramine, Diphenylpyraline, Doxylamine, Belladonna, Codeine, Pseudoephedrine	R05DA	3	3	3	3	3
Chlorpheniramine, Codeine	R05FA	3	3	3	3	3
Carbinoxamine, Codeine	R05FA01	3	3	1	3	3
Carbinoxamine, Chlorpheniramine, Diphenhydramine, Codeine, Pseudoephedrine	R05FA02	3	3	3	3	3
Carbinoxamine, Chlorpheniramine, Dexchlorpheniramine, Diphenylpyraline, Doxylamine, Belladonna, Codeine, Pseudoephedrine	R05X	3	3	3	3	3
Chlorpheniramine, Dimenhydrinate, Diphenhydramine, Scopolamine	R06AA52	3	3	3	3	3
Chlorpheniramine, Meclizine	R06AK	3	3	3	3	3
Desloratadine, Pseudoephedrine	R06AX27	0	1	1	1	1

*A dot (.) in the table means the medication is not scored/listed in the scale. Medications with no scoring in the scales were calculated as 0 point in our study.
†Multiple combination drugs sharing the same ATC codes; only the ingredients with anticholinergic activity listed
‡Combination drugs with redefined score: The score of a combination drug was redefined as the score of the ingredient with the highest score in the combination drug.

Supplementary table 3. Definition of anticholinergic burden categories for anticholinergic burden in the hazard and reference period.

Anticholinergic burden category	Total anticholinergic burden		Used for analyses
	Hazard period	Reference period*	
Category A	1-2	0	➔ 1-2 vs. 0
Category B	0	1-2	
Category C	≥3	0	➔ ≥3 vs. 0
Category D	0	≥3	
Category E	≥3	1-2	➔ ≥3 vs. 1-2
Category F	1-2	≥3	
Category G	0	0	Provide no information
Category H	1-2	1-2	Provide no information
Category I	≥3	≥3	Provide no information

*One randomly selected from four reference periods

†For instance, participants with 1-2 points in hazard and 0 point in reference period were grouped into category A, and those with 0 points in hazard and 1-2 point in reference period were category B. The categories with opposite burden, such as category A and B, were defined as corresponding burden categories for the crossover analysis.

Supplementary table 4. Comorbidities collected for baseline characteristics and used for disease risk score matching.

Comorbidities	ICD-9-CM	ICD-10-CM
Hypertension	401-405	I10-I16
Diabetes	250	E10-E14
Dyslipidaemia	272	E78
Obesity	2780	E66
Heart failure	428	I50
Chronic kidney disease	250.4, 403, 404, 581-583, 585, 586	E102, E112, E132, I12, I13, N03, N04, N05, N18, N19
Chronic liver disease	571, 572, 573.1-573.3	K70, K72-K75
COPD	490-492, 496	J40-J42, J44
Asthma	493	J45
GERD	530.81	K21
Peptic ulcer	530.2, 531-534	K22.1, K25-K28
Dementia (Major neurocognitive disorders)		
Vascular dementia	290.4	F01
Alzheimer's disease	331.0	G30
Parkinson’s dementia	331.89	G31.83
Others or unspecified	290.0, 290.1-290.3, 290.8, 290.9, 294.0, 294.1, 331.1, 331.2	F02, F03, G31.0, G31.1, G31.9
Parkinson's disease	332	G20, G21
Epilepsy	345	G40
Mental illness		
Schizophrenia	295	F20, F21, F25
Depression	296.2, 296.3, 300.4, 309.0, 309.1, 311	F32, F33, F34.1, F43.2
Bipolar disorder	296.0, 296.1, 296.4-296.8	F30, F31
Anxiety	300.0	F41
Alcohol / Drug abuse	305.0, 305.2-305.9	F10-F16, F18, F19, F55
Cancer	140-208, 210-238	C00-C96, D00-D48

* A 3-digit ICD code includes more specific fourth and fifth digits. For example, the ICD-9-CM code “410” includes the wildcard codes “410.xx”.

Supplementary table 5. Medications with anticholinergic activity collected for baseline characteristics.

Medications	ATC code	Medications	ATC code
**Antihistamines**			
Alimemazine	R06AD01	Doxylamine	R03CB53, R05CB10, R06AA
Brompheniramine	R06AB01, R01BA53	Fexofenadine	R06AX26
Buclizine	R06AE01, R06AE51	Homochlorcyclizine	R06AE91
Carbinoxamine	R06AA08, R05FA01	Hydroxyzine	N05BB01
Cetirizine	R06AE07	Ketotifen	R06AX17
Chlorpheniramine	R06AB04, B05XC, N02BE51, R05CA10, R05DB, R05FA, R05FB02, R06AB54	Levocetirizine	R06AE09
Clemastine	R06AA04	Loratadine	R06AX13
Cyproheptadine	R06AX02	Meclizine	R06AE05
Desloratadine	R06AX27	Mequitazine	R06AD07
Dexchlorpheniramine	R06AB02	Phenindamine	R06AX04
Dimenhydrinate	R06AA02	Piprinhydrinate	R06AA07
Diphenhydramine	R06AA02, C05AX, N02CA52, R01AB02, R01AX30	Promethazine	R06AD02
**GI antispasmodics**			
Alverine	A03AX08	Oxapium	A03AB
Atropine	A03BA01, N03AA	Oxyphencyclimine	A03AA01
Belladonna	A03CB02, N02CA72	Pipethanate	A03BA92
Butylscopolamine	A03BB01	Propantheline	A03AB05
Butropium	A03AB91	Scopolamine	A03BB01, R06AE55
Clidinium	A03CA02	Scopolia	A03EA, A07AX
Dicyclomine	A03AA07, A03CC	Timepidium	A03AB19
Glycopyrrolate	A03AB02	Trimebutine	A03AA05
Hyoscine	A03BB01	Trospium	A03AB20
Hyoscyamine	A03BA03, A03CB31	Valethamate bromide	A03AB93
Otilonium bromide	A03AB06		
**Diuretics**			
Furosemide	C03CA01	Triamterene	C03EA01

Hydrochlorothiazide	C02LA51, C03AA03, C03EA01, C09BA02, C09DA01, C09DA03, C09DA04, C09DA06, C09DA07, C09DA08, C09DX01, C09DX03, C09XA52		
**Bronchodilators**			
Glycopyrrolate	R03BB06	Tiotropium	R03AL06, R03BB04
Glycopyrronium	R03AL04, R03AL07, R03AL09	Theophylline	R03DA04, R03DA54, R03DB04
Ipratropium	R03AL01, R03AL02, R03BB01		
**Antipsychotics**			
Amisulpride	N05AL05	Paliperidone	N05AX13
Aripiprazole	N05AX12	Perphenazine	N05AB03
Chlorpromazine	N05AA01	Pimozide	N05AG02
Chlorprothixene	N05AF03	Prochlorperazine	N05AB04
Clozapine	N05AH02	Quetiapine	N05AH04
Flupentixol	N05AF01, N06CA02	Risperidone	N05AX08
Fluphenazine	N05AB02	Sulpiride	N05AL01
Haloperidol	N05AD01	Thioridazine	N05AC02
Levomepromazine/ methotrimeprazine	N05AA02	Trifluoperazine	N05AB06
Lithium	N05AN01	Ziprasidone	N05AE04
Loxapine	N05AH01	Zotepine	N05AX11
Olanzapine	N05AH03	Zuclopenthixol	N05AF05
**Antiemetics / Antivertigo agents**			
Cinnarizine	N07CA02	Diphenidol	N07CA
**Antidepressants**			
Amitriptyline	N06AA09	Fluvoxamine	N06AB08
Bupropion	N06AX12	Imipramine	N06AA02
Citalopram	N06AB04	Maprotiline	N06AA21
Clomipramine	N06AA04	Mirtazapine	N06AX11
Dothiepin	N06AA16	Paroxetine	N06AB05

Doxepin	N06AA12	Sertraline	N06AB06
Escitalopram	N06AB10	Trazodone	N06AX05
Fluoxetine	N06AB03	Venlafaxine	N06AX16
**Muscle relaxants**			
Baclofen	M03BX01	Orphenadrine	M03BC51
Cyclobenzaprine	M03BX08	Pridinol	M03BX03
Methocarbamol	M03BA03	Tizanidine	M03BX02
Orphenadrine	M03BC01		
**Antiepileptics**			
Carbamazepine	N03AF01	Oxcarbazepine	N03AF02
Clonazepam	N03AE01	Valproic acid	N03AG01
**Genitourinary antispasmodics**			
Bromocriptine	G02CB01	Propiverine	G04BD06
Flavoxate	G04BD02	Solifenacin	G04BD08
Oxybutynin	G04BD04	Tolterodine	G04BD07
Pipoxolan	G04BD	Trospium	G04BD09
**Antiparkinson agents**			
Amantadine	N04BB01	Rasagiline	N04BD02
Benztropine	N04AC01	Rotigotine	N04BC09
Biperiden	N04AA02	Selegiline	N04BD01
Entacapone	N04BX02	Trihexyphenidyl/ benzhexol	N04AA01
Pramipexole	N04BC05		
**Antiarrhythmic drugs**			
Disopyramide	C01BA03	Quinidine	C01BA01
**Other combination drugs (containing anticholinergic agents)***			
A02AG	Butinolin, Butylscopolamine, Dicyclomine, Hyoscine, Pipethanate, Scopolamine		
A02AX	Propantheline, Scopolia		
A03CB	Atropine, Butylscopolamine, Hyoscine, Hyoscyamine, Scopolamine		
A09AA	Belladonna, Scopolia		
N04BA02	Carbidopa, Levodopa		
N04BA03	Entacapone, Levodopa		

R01BA51	Brompheniramine, Chlorpheniramine, Belladonna
R01BA52	Carbinoxamine, Chlorpheniramine, Fexofenadine, Loratadine, Triprolidine
R03DA51	Chlorpheniramine, Dimenhydrinate, Theophylline, Scopolamine
R03DA71	Chlorpheniramine, Theophylline
R03DB01	Chlorpheniramine, Diphenhydramine, Theophylline
R05DA	Carbinoxamine, Chlorpheniramine, Diphenhydramine, Theophylline, Belladonna
R05FA02	Carbinoxamine, Chlorpheniramine, Diphenhydramine
R05X	Carbinoxamine, Chlorpheniramine, Dexchlorpheniramine, Diphenylpyraline, Doxylamine, Belladonna
R06AA52	Chlorpheniramine, Dimenhydrinate, Diphenhydramine, Scopolamine
R06AK	Chlorpheniramine, Meclizine

*Multiple combination drugs sharing the same ATC codes; only the ingredients with anticholinergic activity listed

Supplementary table 6. Covariates included for time-varying confounders adjustment in sensitivity analysis.

Time-varying covariates	ICD-9-CM	ICD-10-CM
Acute infections		
Pneumonia / Influenza / Upper respiratory tract infection	466.0, 480-488, 507	J09-J22, J44.0, J69
Bloodstream infection (Bacteraemia / Sepsis)	003.1, 038, 036.2, 112.5, 790.7	A40, A41, B37.7, R65, R78.81, A39.2
Acute urinary tract infection	590, 595, 599.0	N10, N12, N13.6, N15.1, N28.8, N30.0, N39.0
Endocarditis	391.1, 421, 112.81, 036.42, 098.84, 115.04, 115.14, 115.94, 424.9, 093.2	I01.1, I33, I38, I39, B37.6, A39.51, B33.21, A52.03, A54.83
Myocarditis	422, 036.43, 093.82, 391.2	I01.2, I41, I40, A39.52, B33.22
Acute kidney injury	572.4, 584	N17, K76.7

‡ A 3-digit ICD code includes more specific fourth and fifth digits. For example, the ICD-9-CM code “410” includes the wildcard codes “410.xx”.

Supplementary table 7. Different scales for anticholinergic burden measurement in sensitivity analysis.

Abbrev.	Full name	Country, Year	Author	Total drugs listed (n)*	Drugs scored >0† (n)	Scoring levels	Methods to determine anticholinergic scores
ACB	Anticholinergic Cognitive Burden Scale	USA, 2008	Boustani *et al.*	NA	88	0-3	• Expert opinions<br>• Literature review: SAA, *in vitro* affinity to muscarinic receptor, BBB permeability, adverse events
ADS	Anticholinergic Drug Scale	USA, 2002/2006	Carnahan *et al.*	520	117	0-3	• Expert opinions<br>• SAA assessment<br>• Adverse events assessment<br>• Based on existing scales
GABS	German Anticholinergic Burden Scale	Germany, 2018	Kiesel *et al.*	504	151	0-3	• Expert opinions<br>• Literature review: SAA, adverse effects, BBB permeability<br>• Based on existing scales
m-ACB	Modified Anticholinergic Cognitive Burden Scale	Korea, 2018	Ah *et al.*	169	169	1-3	• Literature review: SAA, adverse effects, BBB permeability<br>• Based on existing scale
KABS	Korean Anticholinergic Activity Scale	Korea, 2019	Jun *et al.*	494	138	0-3	• Expert opinions<br>• Literature review: SAA, adverse effects, BBB permeability<br>• Based on existing scales

SAA=Serum anticholinergic activity; BBB=blood-brain barrier

*NA in total drugs included: Medications with none or limited anticholinergic activity (= 0 point) were not published in the scale or article.

†The number of medications determined to have anticholinergic activity in any potency level (low to high anticholinergic potency, score > 0 point). The medications recognised to be "none or limited anticholinergic activity (= 0 point)" were not included in the number of drugs scored in this table.

Supplementary table 8. Characteristics in baseline periods of current cases and future cases in the main analysis

Baseline period	Current cases (n=248,579)		Future cases (n=248,579)		SMD
Age, mean (SD)	78.3	(0.02)	78.3	(0.02)	0.00
Male, n (%)	133158	(53.6)	133158	(53.6)	0.00
**Comorbidities, n (%)**					
Hypertension	155411	(62.5)	150933	(60.7)	0.04
Heart failure	26627	(10.7)	20386	(8.2)	0.09
Diabetes mellitus	81330	(32.7)	78715	(31.7)	0.02
Dyslipidaemia	60442	(24.3)	59667	(24.0)	0.01
Chronic kidney disease	49676	(20.0)	46234	(18.6)	0.04
Chronic liver disease	16997	(6.8)	15832	(6.4)	0.02
Asthma	17661	(7.1)	15898	(6.4)	0.03
COPD	40677	(16.4)	36500	(14.7)	0.05
GI ulcer / GERD	45864	(18.5)	42288	(17.0)	0.04
Dementia	27290	(11.0)	25113	(10.1)	0.03
Parkinson's disease	9459	(3.8)	8929	(3.6)	0.01
Epilepsy	2103	(0.9)	1842	(0.7)	0.01
Mental illness*	26868	(10.8)	25719	(10.4)	0.02
Schizophrenia	1145	(0.5)	1046	(0.4)	0.01
Osteoporosis	18129	(7.3)	17205	(6.9)	0.01
Alcohol / Drug abuse	352	(0.1)	279	(0.1)	0.01
Solid tumour	15071	(6.1)	13884	(5.6)	0.02
Hematologic cancer	1126	(0.5)	952	(0.4)	0.01
**Use of medications with anticholinergic activity, n (%)**					
Antihistamines	171518	(69.0)	163255	(65.7)	0.07
GI antispasmodics	102000	(41.0)	95122	(38.3)	0.06
Diuretics	84003	(33.8)	72535	(29.2)	0.10
Bronchodilators	68181	(27.4)	58966	(23.7)	0.09
Antiemetics / Antivertigo agents	51136	(20.6)	44381	(17.9)	0.07
Antipsychotics	44122	(17.8)	36973	(14.9)	0.08
Antidepressants	27874	(11.2)	26221	(10.6)	0.02
Antiepileptics	22600	(9.1)	20509	(8.3)	0.03
Muscle relaxants	20916	(8.4)	19546	(7.9)	0.02

GU antispasmodics	20614	(8.3)	19665	(7.9)	0.01
Antiparkinson agents	13550	(5.5)	12683	(5.1)	0.02
Antiarrhythmic drugs	219	(0.1)	208	(0.1)	0.00

COPD=chronic obstructive pulmonary disease; GI=gastrointestinal; GERD=gastroesophageal reflux disease; GU=genitourinary; SMD=standardized mean difference; SD=standard deviation

*Depression / Bipolar disorder/ Anxiety

†A future-case control was selected by 1:1 matching with a current case on age and sex. The demographic characteristics shown in the table are the results after matching.

Supplementary table 9. Characteristics in hazard and reference periods of current cases and future cases in the main analysis

	Current cases (n=248,579)					Future cases (n=248,579)					
	**Hazard period**		**Reference period**		**SMD**	**Hazard period**		**Reference period**		**SMD**
**Use of medications with anticholinergic activity, n (%)**										
Antihistamines	74056	(30.5)	53939	(22.2)	0.19	55798	(23.0)	51265	(21.1)	0.05
Diuretics	47002	(19.4)	34685	(14.3)	0.14	32135	(13.2)	27478	(11.3)	0.06
GI antispasmodics	33241	(13.7)	22503	(9.3)	0.14	23298	(9.6)	21450	(8.8)	0.03
Bronchodilators	28160	(11.6)	17999	(7.4)	0.14	18008	(7.4)	15317	(6.3)	0.04
Antipsychotics	19893	(8.2)	13634	(5.6)	0.1	13205	(5.4)	11012	(4.5)	0.04
Antiemetics / Antivertigo agents	19001	(7.8)	10943	(4.5)	0.14	10873	(4.5)	9790	(4.0)	0.02
Antidepressants	13746	(5.7)	12237	(5.0)	0.03	11049	(4.6)	10270	(4.2)	0.02
Antiepileptics	11448	(4.7)	9644	(4.0)	0.04	8825	(3.6)	7934	(3.3)	0.02
Antiparkinson agents	8694	(3.6)	7626	(3.1)	0.02	6537	(2.7)	5938	(2.5)	0.02
GU antispasmodics	6872	(2.8)	5978	(2.5)	0.02	5497	(2.3)	4967	(2.1)	0.02
Muscle relaxants	5767	(2.4)	4440	(1.8)	0.04	4384	(1.8)	3883	(1.6)	0.02
Antiarrhythmic drugs	93	(0.0)	76	(0.0)	0	66	(0.0)	65	(0.0)	0

SMD=standardized mean difference

Supplementary table 10. Results of subgroup analyses matching current cases and future cases on diagnoses of individual cardiovascular events.

Analyses	Patient No		Odds ratio (95% CI)					
	Current cases*	Future cases*	1-2 vs. 0		≥3 vs. 0		≥3 vs. 1-2	
**Main analysis**	248,579	248,579	1.38	(1.34 to 1.42)	2.03	(1.98 to 2.09)	1.48	(1.44 to 1.52)
**Subgroup analysis**								
Myocardial infarction	31,348	31,348	1.34	(1.24 to 1.45)	2.20	(2.05 to 2.37)	1.64	(1.52 to 1.77)
Ischaemic Stroke	72,519	72,519	1.42	(1.35 to 1.50)	2.05	(1.96 to 2.15)	1.44	(1.37 to 1.52)
Haemorrhage strokes	31,511	31,511	1.26	(1.16 to 1.37)	1.62	(1.50 to 1.74)	1.28	(1.18 to 1.40)
Dysrhythmias	62,203	62,203	1.41	(1.33 to 1.49)	2.17	(2.06 to 2.28)	1.54	(1.46 to 1.63)
Conduction disorders	6,253	6,253	1.29	(1.08 to 1.53)	1.82	(1.56 to 2.13)	1.42	(1.19 to 1.68)
Syncope	6,636	6,636	1.19	(1.00 to 1.42)	1.84	(1.58 to 2.15)	1.55	(1.31 to 1.83)
Cardiovascular death	33,081	33,081	1.43	(1.32 to 1.55)	2.43	(2.25 to 2.63)	1.70	(1.58 to 1.83)

Supplementary table 11. Frequencies of participants in different anticholinergic burden categories in subgroup analysis.

No of participants		Myocardial infarction		Ischaemic stroke		Haemorrhage strokes		Dysrhythmias		Conduction disorders		Syncope		Cardiovascular death	
		Current cases	Future cases	Current cases	Future cases	Current cases	Future cases	Current cases	Future cases	Current cases	Future cases	Current cases	Future cases	Current cases	Future cases
**Total participant**		31,348	31,348	72,519	72,519	31,511	31,511	62,203	62,203	6,253	6,253	6,636	6,636	33,081	33,081
**Participants in different burden categories**															
Hazard period	Reference Period														
1-2	0	2,170	1,747	5,058	3,787	1,950	1,571	4,293	3,443	444	370	431	372	2,192	1,819
0	1-2	1,135	1,301	2,551	2,948	1,246	1,348	1,960	2,464	231	259	223	249	963	1,249
≥3	0	4,011	2,370	9,363	6,005	3,463	2,452	8,819	5,516	833	505	947	658	4,426	2,483
0	≥3	1,544	1,930	3,739	4,659	1,838	2,019	2,781	3,514	360	389	334	407	1,180	1,507
≥3	1-2	2,487	1,595	4,607	3,007	1,639	1,187	5,751	3,659	479	322	552	359	3,274	1,824
1-2	≥3	1,437	1,585	2,980	3,039	1,153	1,152	3,227	3,415	310	303	322	345	1,520	1,518
0	0	9,360	11,682	24,726	30,298	12,795	14,539	14,000	19,416	1,665	2,194	1,676	2,211	8,487	11,682
1-2	1-2	3,319	3,896	7,019	7,775	2,732	2,990	6,700	8,028	705	787	657	757	3,292	4,290
≥3	≥3	5,885	5,242	12,476	11,001	4,695	4,253	14,672	12,748	1,226	1,124	1,494	1,278	7,747	6,709

Supplementary table 12. Results of primary analysis and sensitivity analyses using different scales for anticholinergic burden measurement.

	Odds ratio (95% CI)									
	Primary analysis		Sensitivity analysis							
	ACB		ADS		GABS		m-ACB		KABS	
**Score 1-2 vs. 0**										
Case-crossover	1.86	(1.83 to 1.90)	1.93	(1.89 to 1.97)	1.95	(1.92 to 1.99)	1.88	(1.84 to 1.91)	1.97	(1.93 to 2.01)
Control-crossover	1.35	(1.33 to 1.38)	1.38	(1.35 to 1.41)	1.38	(1.35 to 1.40)	1.38	(1.35 to 1.40)	1.40	(1.37 to 1.43)
CCTC*	1.38	(1.34 to 1.42)	1.40	(1.36 to 1.44)	1.42	(1.38 to 1.46)	1.36	(1.32 to 1.40)	1.41	(1.37 to 1.45)
**Score 3+ vs. 0**										
Case-crossover	2.91	(2.86 to 2.96)	3.25	(3.19 to 3.31)	3.47	(3.41 to 3.54)	3.02	(2.96 to 3.07)	2.95	(2.90 to 3.00)
Control-crossover	1.43	(1.41 to 1.46)	1.48	(1.45 to 1.50)	1.54	(1.51 to 1.57)	1.45	(1.43 to 1.48)	1.42	(1.40 to 1.45)
CCTC*	2.03	(1.98 to 2.09)	2.20	(2.15 to 2.26)	2.25	(2.19 to 2.31)	2.07	(2.02 to 2.13)	2.08	(2.03 to 2.13)
**Score 3+ vs. 1-2**										
Case-crossover	1.56	(1.53 to 1.59)	1.68	(1.65 to 1.71)	1.78	(1.74 to 1.81)	1.61	(1.58 to 1.64)	1.50	(1.47 to 1.53)
Control-crossover	1.06	(1.04 to 1.08)	1.07	(1.05 to 1.09)	1.12	(1.10 to 1.14)	1.06	(1.04 to 1.08)	1.01	(0.99 to 1.04)
CCTC*	1.48	(1.44 to 1.52)	1.57	(1.53 to 1.61)	1.59	(1.54 to 1.63)	1.52	(1.48 to 1.56)	1.48	(1.43 to 1.52)

ADS= Anticholinergic Drug Scale; GABS= the German Anticholinergic Burden Scale; m-ACB= the Modified Anticholinergic Cognitive Burden Scale; KABS= Korean Anticholinergic Activity Scale; CCTC= Case-case-time-control.

Supplementary table 13. Results of sensitivity analyses excluding patients with special conditions in baseline period.

Analyses	Patient No		Odds ratio (95% CI)					
	Current cases*	Future cases*	1-2 vs. 0		≥3 vs. 0		≥3 vs. 1-2	
**Main analysis**	248,579	248,579	1.38	(1.34 to 1.42)	2.03	(1.98 to 2.09)	1.48	(1.44 to 1.52)
**Sensitivity analysis: Matching on individual outcome**								
Exclusion of patients with CV drugs†	114,838	116,223	1.48	(1.40 to 1.55)	1.99	(1.91 to 2.06)	1.35	(1.28 to 1.42)
Exclusion of patients with CV death	210,470	210,470	1.41	(1.36 to 1.45)	2.15	(2.09 to 2.21)	1.53	(1.48 to 1.58)
Exclusion of patients with myocarditis or endocarditis	241,444	241,746	1.38	(1.35 to 1.42)	2.03	(1.98 to 2.08)	1.46	(1.42 to 1.51)
Exclusion of patients with a record of hospitalization	169,964	183,862	1.36	(1.32 to 1.41)	2.04	(1.98 to 2.10)	1.50	(1.45 to 1.55)
Exclusion of patients with infectious diseases	84,626	95,740	1.37	(1.30 to 1.44)	1.96	(1.87 to 2.07)	1.43	(1.35 to 1.52)
Exclusion of patients with influenza	234,078	235,075	1.38	(1.34 to 1.42)	2.02	(1.97 to 2.07)	1.46	(1.42 to 1.51)

CV=cardiovascular

* Patient numbers in the sensitivity analyses were the numbers of patients after exclusion.
† Infectious diseases including upper respiratory tract infection, pneumonia, influenza, urinary tract infection and bloodstream infection.

Supplementary table 14. Results of primary analysis and sensitivity analyses of the crude case-crossover and case-time-control analysis.

Analyses comparing total anticholinergic burden	Odds ratio (95% CI)			
	**Primary analysis**	**Sensitivity analysis**		
	**Case-case-time-control (CCTC) analysis**	**Crude case crossover analysis**	**Case-time-control (CTC) analysis with DRS measured on covariates within -0 to -180 days (baseline period)**	**Case-time-control (CTC) analysis with DRS measured on covariates within -180 to -540 days**
	(N = 248,579)	(N =317,446)	(N =263,165)	(N =308,706)
**1-2 vs. 0**				
Case crossover	1.86 (1.83 to 1.90)	1.83 (1.80 to 1.86)	1.88 (1.85 to 1.92)	1.82 (1.79 to 1.86)
Control crossover	1.35 (1.33 to 1.38)	-	1.18 (1.16 to 1.21)	1.19 (1.17 to 1.22)
Case/control ratio	1.38 (1.34 to 1.42)	-	1.59 (1.55 to 1.64)	1.53 (1.49 to 1.57)
**≥3 vs. 0**				
Case crossover	2.91 (2.86 to 2.96)	2.93 (2.89 to 2.98)	2.97 (2.92 to 3.03)	2.96 (2.91 to 3.01)
Control crossover	1.43 (1.41 to 1.46)	-	1.21 (1.19 to 1.23)	1.24 (1.21 to 1.26)
Case/control ratio	2.03 (1.98 to 2.09)	-	2.46 (2.39 to 2.52)	2.39 (2.34 to 2.45)
**≥3 vs. 1-2**				
Case crossover	1.56 (1.53 to 1.59)	1.60 (1.57 to 1.63)	1.58 (1.55 to 1.61)	1.62 (1.60 to 1.65)
Control crossover	1.06 (1.04 to 1.08)	-	1.03 (1.00 to 1.05)	1.04 (1.02 to 1.06)
Case/control ratio	1.48 (1.44 to 1.52)	-	1.54 (1.50 to 1.59)	1.56 (1.52 to 1.60)

DRS= disease risk score

Supplementary table 15. Frequencies of participants in different anticholinergic burden categories in primary analysis and sensitivity analyses of the crude case-crossover and case-time-control analysis.

No of participants		Odds ratio (95% CI)					
		Primary analysis		Sensitivity analysis			
		Case-case-time-control (CCTC) analysis (N = 248,579)		Crude case crossover analysis (N =317,446)		Case-time-control (CTC) analysis (N =263,165)	
**Participants in different burden categories**							
**Hazard period**	**Reference Period**	**Current cases**	**Future case**	**Eligible participants**		**Cases**	**External controls**
1-2	0	17,603	14,247	22,284		10,958	19,151
0	1-2	8,507	10,436	10,995		9,309	9,252
≥3	0	33,174	21,363	41,558		17,411	35,901
0	≥3	12,210	15,005	15,181		14,346	12,873
≥3	1-2	19,432	12,703	25,056		9,796	18,487
1-2	≥3	11,448	11,924	14,416		9,591	10,724
0	0	71,442	88,773	92,331		133,546	84,749
1-2	1-2	24,889	29,578	32,174		26,777	25,850
≥3	≥3	49,874	44,550	63,451		31,431	46,178
**Participants in each corresponding burden categories**		**Higher burden in hazard period†**	**Higher burden in reference period**	**Higher burden in hazard period†**	**Higher burden in reference period**	**Higher burden in hazard period†**	**Higher burden in reference period**
**1-2 vs. 0**							
Case crossover		17,603	8,507	22,284	10,995	10,958	9,309
Control crossover		14,247	10,436	-	-	19,151	9,252
**≥3 vs. 0**							
Case crossover		33,174	12,210	41,558	15,181	17,411	14,346
Control crossover		21,363	15,005	-	-	35,901	12,873
**≥3 vs. 1-2**							
Case crossover		19,432	11,448	25,056	14,416	9,796	9,796
Control crossover		12,703	11,924	-	-	18,487	10,724

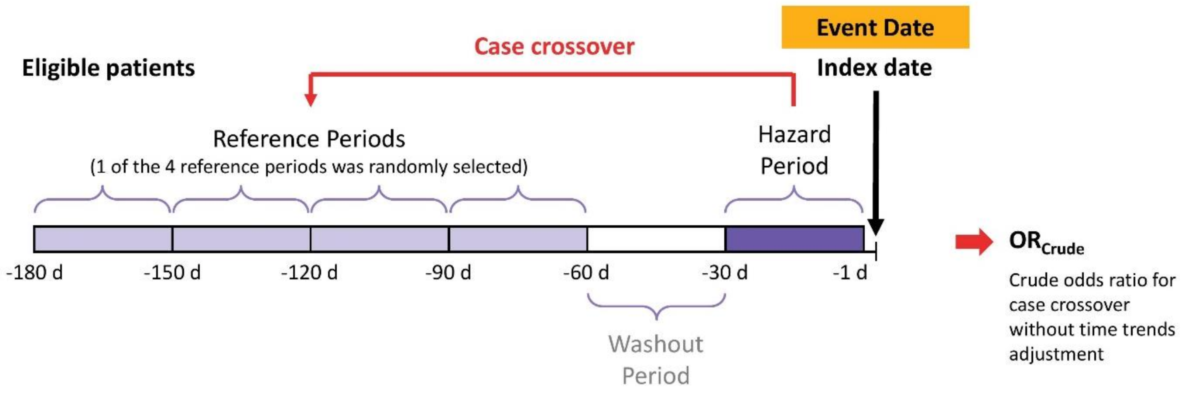

Supplementary figure 1. Illustration for study design of the crude case-crossover analysis.

A crude case-crossover analysis without adjustment of time trends among all eligible participants

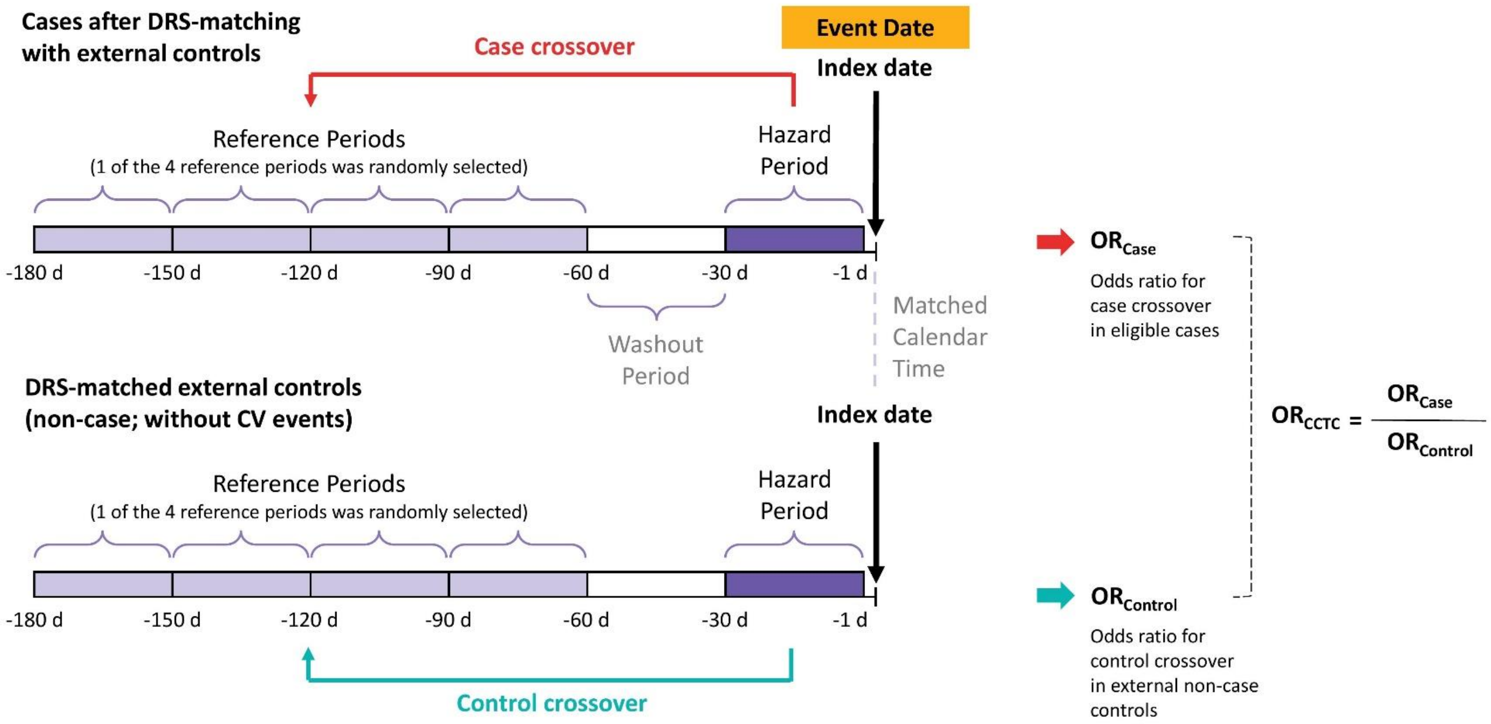

Supplementary figure 2. Illustrations for study design of the case-time-control (CTC) analysis.

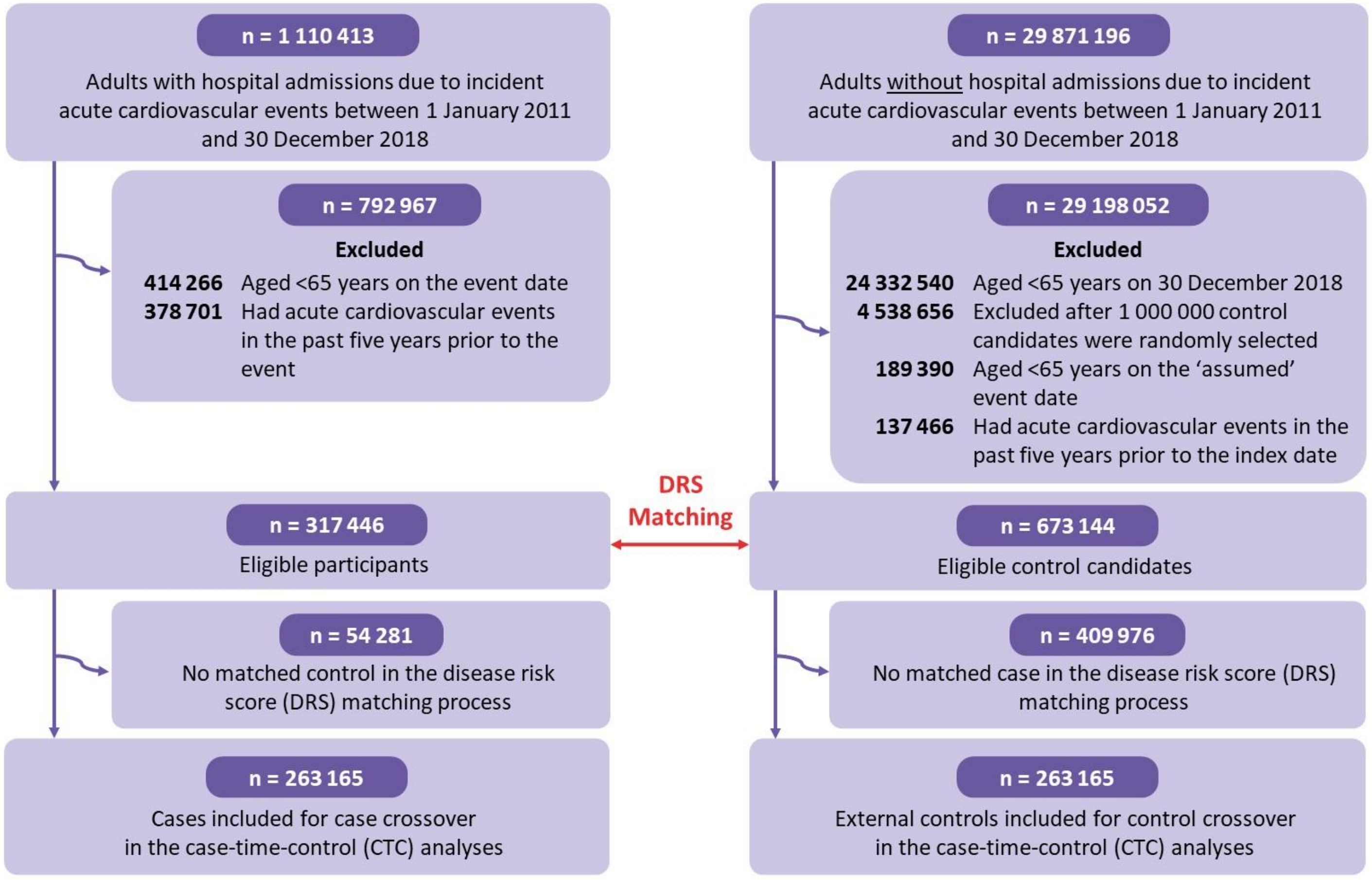

Supplementary figure 3. Flowchart for selection of cases and external controls in the case-time-control (CTC) analysis.

(a) Current cases in case crossover in the CCTC analysis

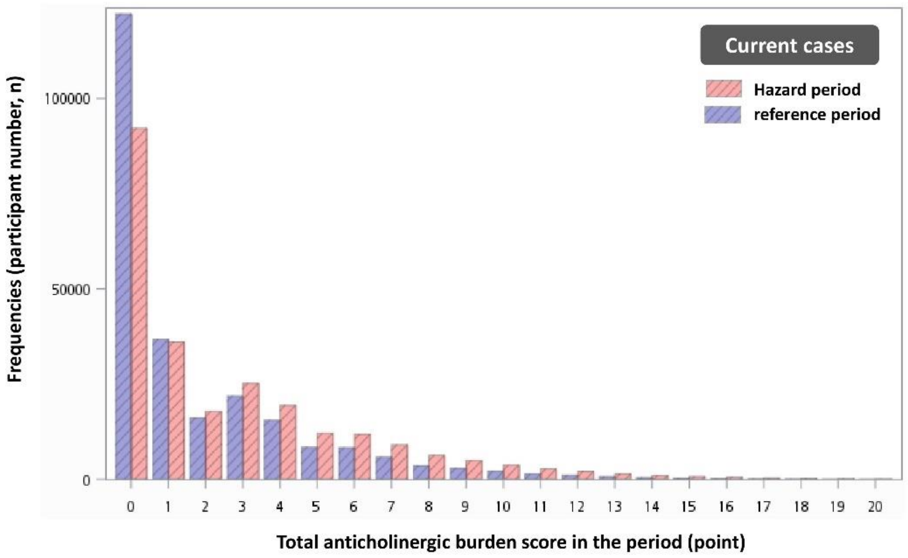

(b) Future cases in control crossover in the CCTC analysis

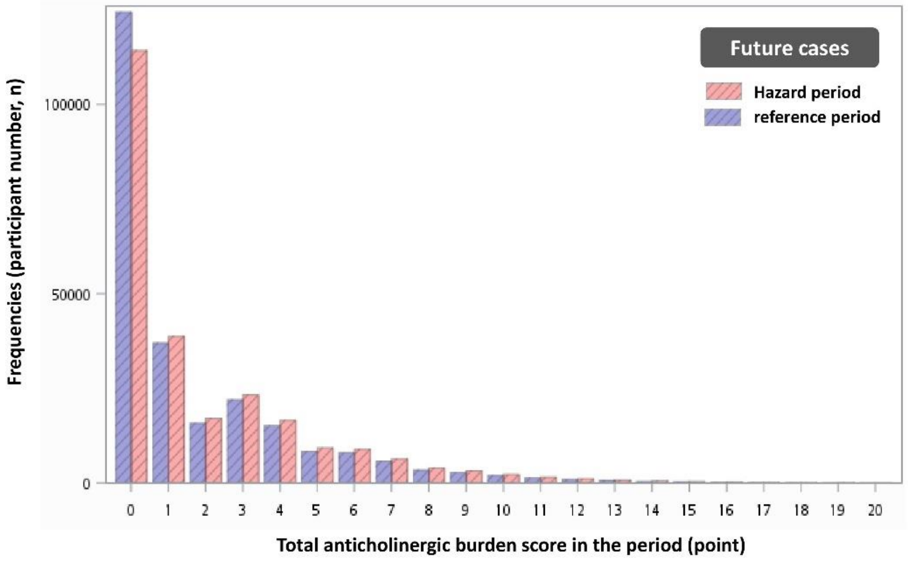

Supplementary figure 4. Distribution of total anticholinergic burden in hazard and reference periods among current cases and future cases in the case-case-time-control analysis.

(a) Histogram for frequencies of current cases with different levels of total anticholinergic burden. (b) Histogram for frequencies of future cases with different levels of total anticholinergic burden.

*The x-axis of the histograms is the total score in hazard or reference period. The percentage in the y-axis is the frequencies (patient numbers). Bars in red: hazard period; bars in blue: reference period.

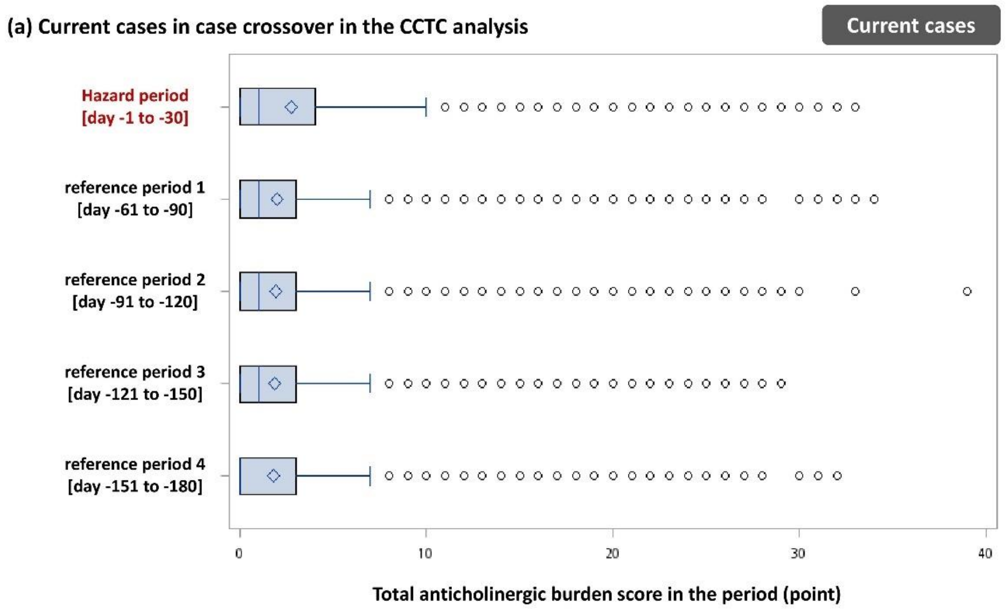

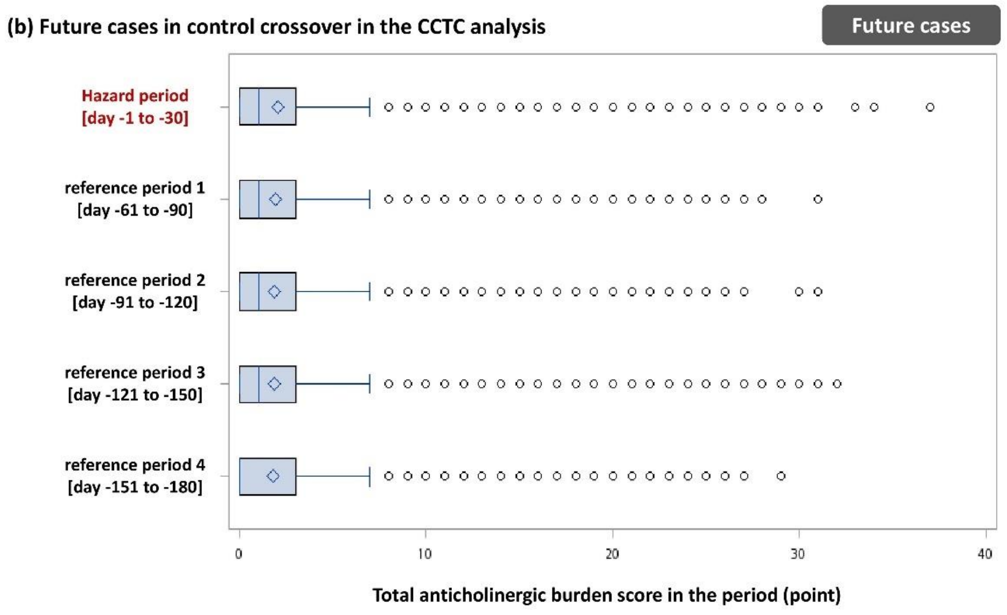

Supplementary figure 5. Box and whisker plot for total anticholinergic burden in hazard and reference periods among current cases and future cases in the case-case-time-control analysis.

(a) Box and whisker plot for total anticholinergic burden in each period among current cases. (b) Box and whisker plot for total anticholinergic burden in each period among future cases.
†The diamond mark in the box and whisker plots represents the mean of the total anticholinergic burden among all current cases or future cases.

(a)

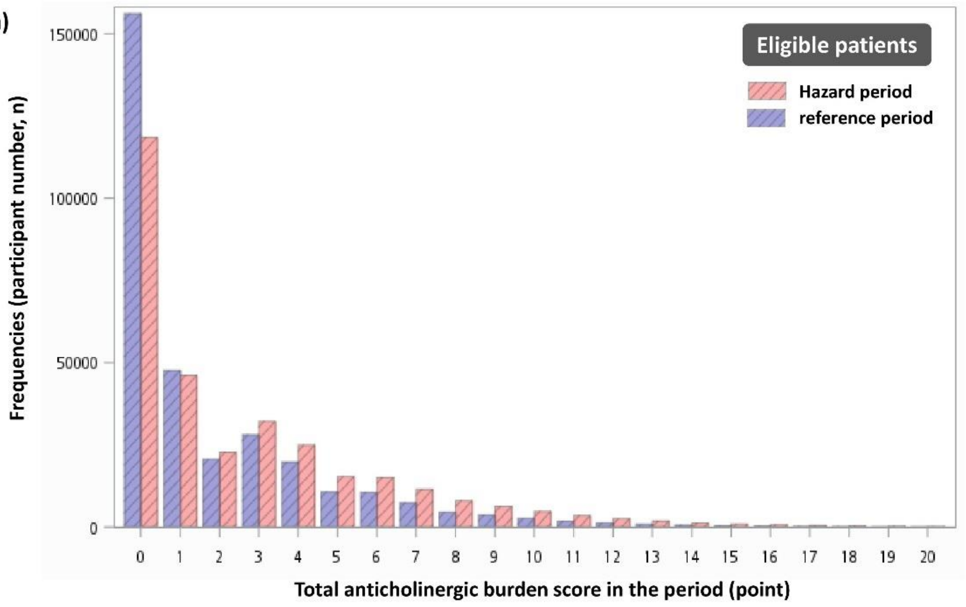

(b)

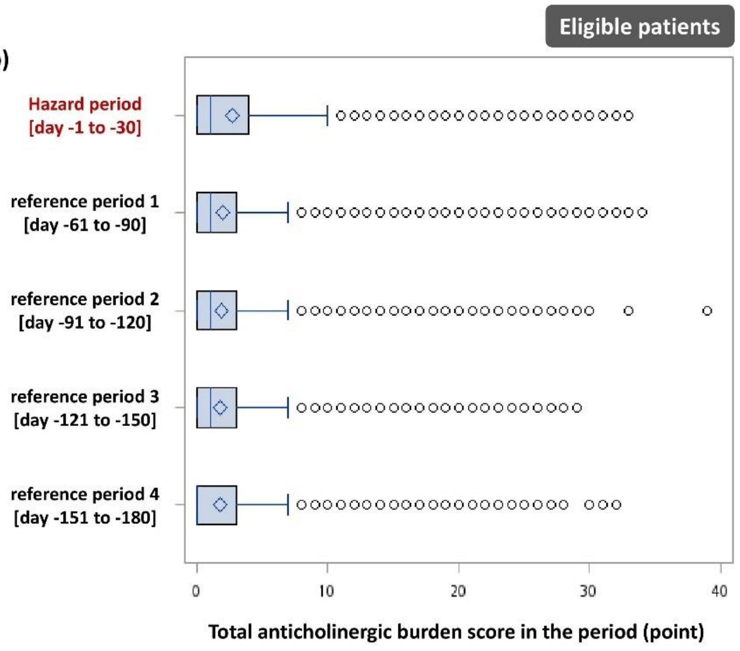

Supplementary figure 6. Distribution and box and whisker plot for total anticholinergic burden in hazard and reference periods among eligible patients in the crude case-crossover analysis.

(a) Histogram for frequencies of all eligible participants with different levels of total anticholinergic burden.
(b) Box and whisker plot for total anticholinergic burden in each period among all eligible participants

*The x-axis of the histograms is the total score in hazard or reference period. The percentage in the y-axis is the frequencies (patient numbers). Bars in red: hazard period; bars in blue: reference period.
†The diamond mark in the box and whisker plots represents the mean of the total anticholinergic burden.

(a) Cases in case crossover in the CTC analysis

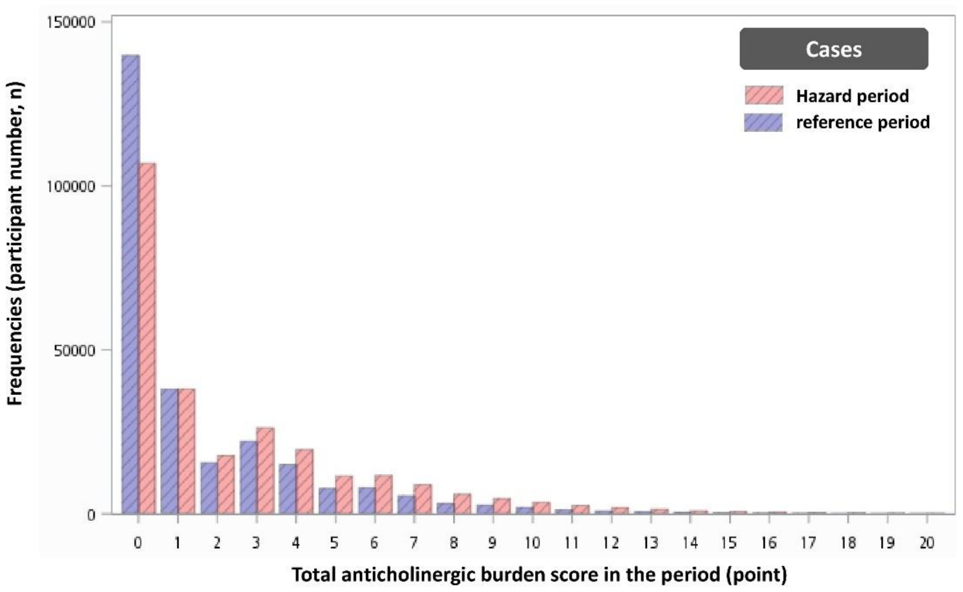

(b) External controls in control crossover in the CTC analysis

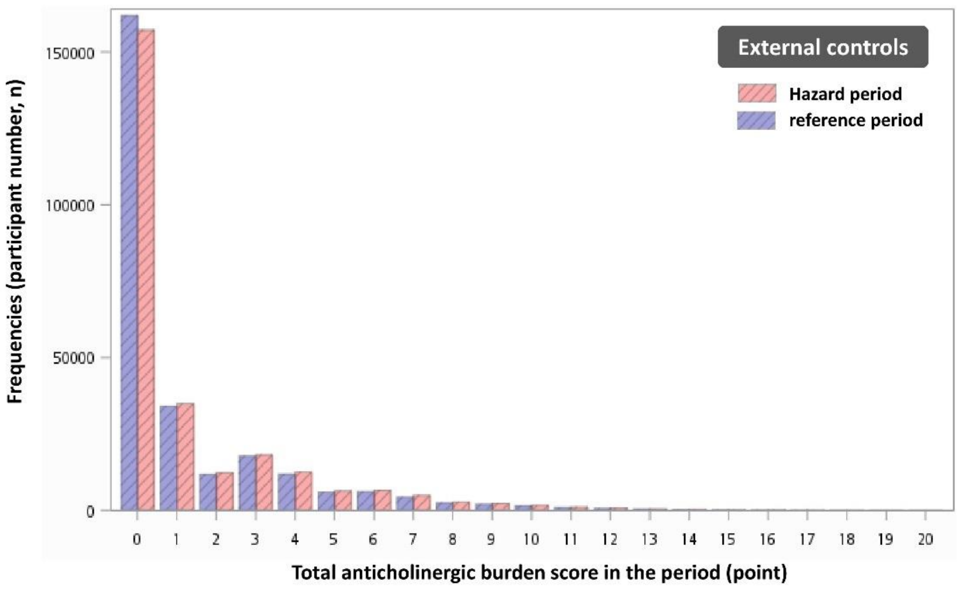

Supplementary figure 7. Distribution of total anticholinergic burden in hazard and reference periods among cases and external controls in the case-time-control analysis.

(a) Histogram for frequencies of cases with different levels of total anticholinergic burden. (b) Histogram for frequencies of external controls with different levels of total anticholinergic burden.

*The x-axis of the histograms is the total score in hazard or reference period. The percentage in the y-axis is the frequencies (patient numbers). Bars in red: hazard period; bars in blue: reference period.

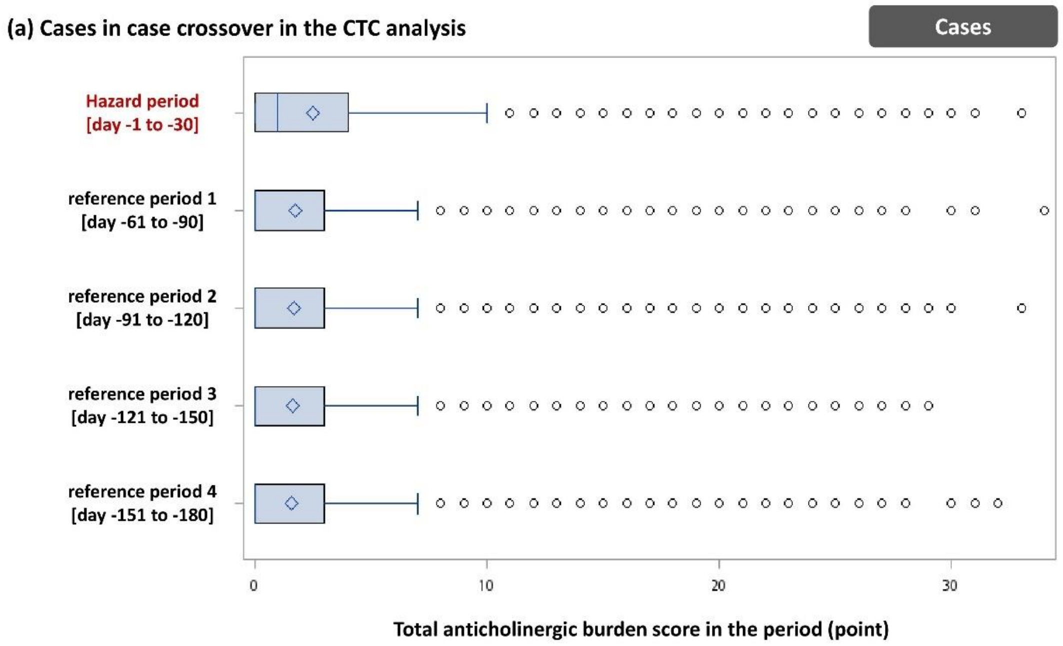

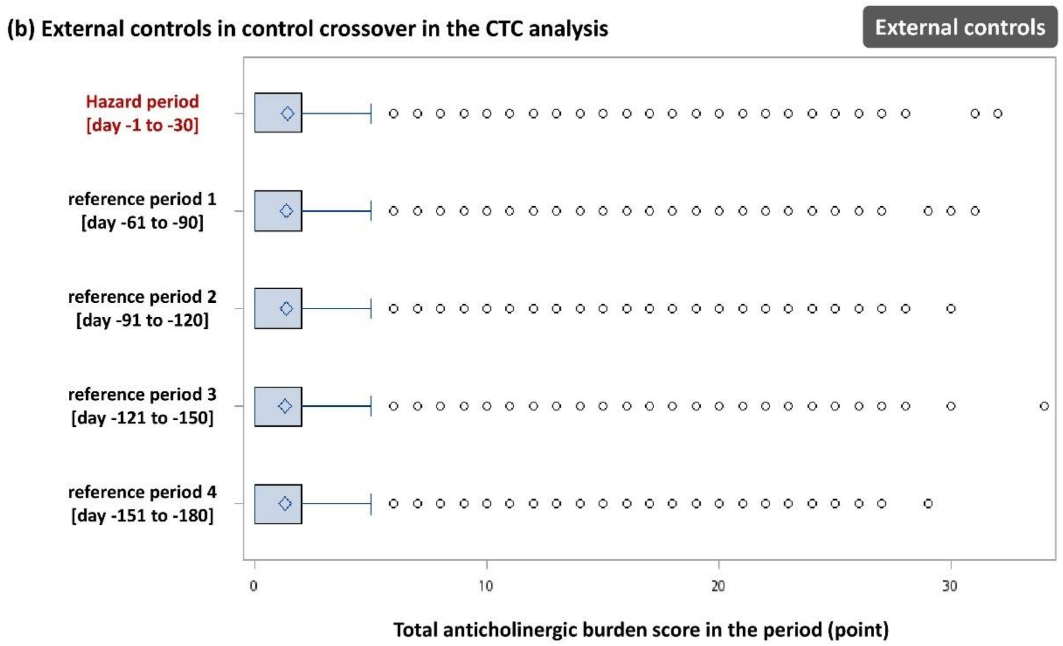

Supplementary figure 8. Box and whisker plot for total anticholinergic burden in hazard and reference periods among cases and external controls in the case-time-control analysis.

(a) Box and whisker plot for total anticholinergic burden in each period among cases. (b) Box and whisker plot for total anticholinergic burden in each period among external controls.
†The diamond mark in the box and whisker plots represents the mean of the total anticholinergic burden among all cases or external controls.
